# Ligand effect of cyclometallated iridium(iii) complexes on *N*-alkylation of amines in hydrogen borrowing reactions†

**DOI:** 10.1039/d3ra07184g

**Published:** 2023-10-31

**Authors:** Yi-Sheng Chen, Siang-Yu Chiu, Chia-Ying Li, Tsun-Ren Chen, Jhy-Der Chen

**Affiliations:** a Department of Applied Chemistry, National Ping Tung University Pingtong City Taiwan trchen@mail.nptu.edu.tw; b Department of Chemistry, Chung Yuan Christian University Chung-Li Taiwan R.O.C

## Abstract

Dinuclear iridium complexes with the general formula (C^N)_2_Ir(μ-Cl)_2_Ir(C^N)_2_ (C^N = bidentate ligand with carbon and nitrogen donor atoms) were prepared and used in catalytic systems for *N*-alkylation of amines through the hydrogen borrowing pathway. Triphenylphosphine derivatives were used as auxiliary in catalytic systems to provide excellent conversion of amines to *N*-alkylation products in yields ranging from 57% to 100%. The catalytic ability of the catalyst depends on the structure of its coordination ligands, including bidentate ligands (C^N) and triphenylphosphine derivatives. These catalytic systems adopt an environmentally friendly and sustainable reaction process through a hydrogen self-transfer strategy, using readily available alcohols as alkylating agents without the need for bases, solvents, and other additives, showing potential in the synthetic and pharmaceutical industries.

## Abstract 2

Dinuclear iridium complexes with the general formula (C^N)_2_Ir(μ-Cl)_2_Ir(C^N)_2_ (C^N = bidentate ligand with carbon and nitrogen donor atoms) were prepared and used in catalytic systems for *N*-alkylation of amines through the hydrogen borrowing pathway.

## Graphical abstract

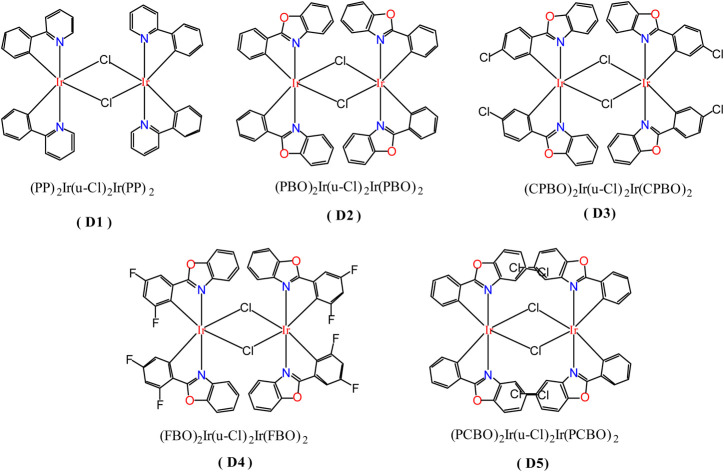

## Introduction

*N*-Alkylation is an important process in the chemical and pharmaceutical industries because nitrogen-containing organic moieties are important building blocks that play crucial roles in bioactive compounds.^1–3^ To date, alkylation of amines with alkyl halides has been widely used industrially to produce target molecules with nitrogen backbones.^4–8^ This reaction is generally fast but also has significant disadvantages such as the introduction of tedious purification procedures, the use of toxic reagents that are harmful to human health, and environmentally unfriendly stoichiometric waste.

Therefore, there is an urgent need to develop efficient and environmentally friendly amine alkylation (*N*-alkylation) processes using harmless and readily available starting materials; in order to overcome the problems caused by halide-based *N*-alkylation problem, many studies have been conducted, including Buchwald–Hartwig coupling,^9^ hydroamination,^10^ and Ullmann reaction.^11^ Recently, a more robust and sustainable approach has attracted attention, employing a catalytic “hydrogen borrowing (HB)” strategy by using less toxic and more readily available alcohols as alkylating agents to form new C–N bonds.

**Scheme 1:**
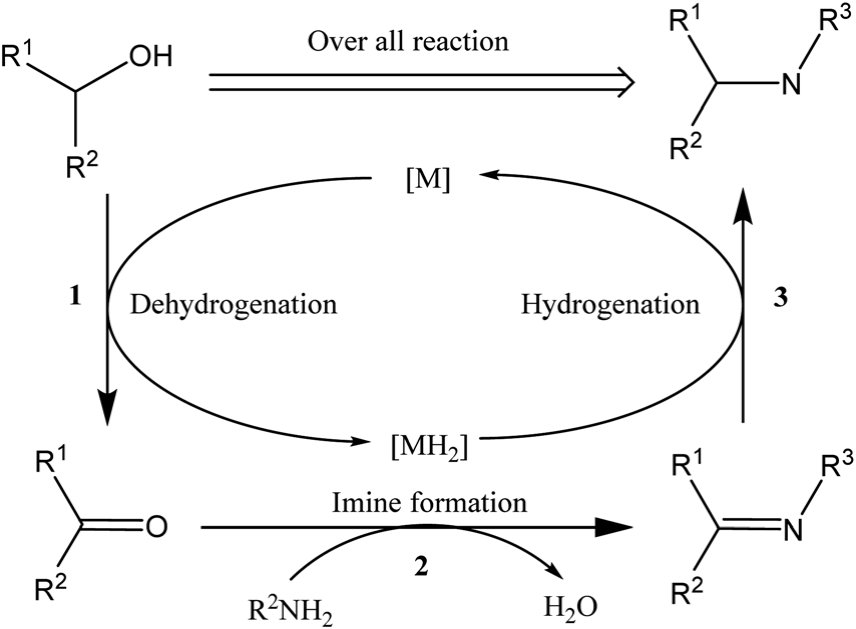
*N*-Alkylation by hydrogen borrowing.

Scheme 1 shows the general mechanism of the hydrogen borrowing strategy,^12,13^ involving the following steps: (1) the two hydrogens of the alcohol are transferred to the catalyst metal, and the alcohol is converted into a more reactive and highly electrophilic carbonyl compound, (2) the carbonyl compound undergoes nucleophilic addition of amine and elimination of H_2_O to form an imine, and (3) in the final stage catalytic hydrogenation of the imine occurs by transferring borrowed hydrogen to form an alkylated amine. This strategy has attracted widespread attention because the only byproduct of the process is water, providing a sustainable approach with high atomic efficiency.^14,15^

Until now, a variety of catalysts have been discovered for the *N*-alkylation of amines using alcohols as alkylating agents, the most common of which are the iridium^16,17^ and ruthenium^18,19^ complexes, but many other metals, such as cobalt,^20,21^ nickel,^22^ rhodium,^23^ palladium,^24,25^ and rhenium,^26^ complexes, have also been explored. Most of these studies report the development of a series of substrates, many of which exhibit good to excellent catalytic ability for *N*-alkylation, but most require the use of bases, solvents, and other additives, which limits the development of truly green processes.

Some groups, such as Shimizu *et al.* (2013),^27^ Albrecht *et al.* (2017),^28^ Wang *et al.* (2019),^29^ and Özdemir *et al.* (2015),^30^ developed base-free *N*-alkylation of amines *via* the BH reaction, but still required solvents or other additives. These reports mainly focus on the use of benzyl alcohol as an alkylating reagent for the alkylation of aniline or benzylamine.

It is worth mentioning that, in 2017, Williams and co-workers reported a method for the *N*-alkylation of amines using ruthenium complexes as homogeneous catalysts without requiring solvents and bases,^31^ indicating that various amines have been successfully synthesized by benzyl alcohol alkylation. Under neat conditions, there are good to excellent conversions and yields, but alcohols other than benzyl alcohol are not described in this article, nor are amines other than primary amines.

Here, we report a series of catalytic systems comprising dinuclear iridium complexes as precursors and triphenylphosphine derivatives as auxiliary ligands for the *N*-alkylation of amines *via* a hydrogen borrowing process. These systems exhibit excellent *N*-alkylation performance for a variety of substrates under one-pot conditions without the need for bases, solvents, and other additives. The role of C^N ligands and triphenylphosphine derivatives in determining the catalytic efficiency of C–N bond formation is discussed in detail.

## Results and discussion

### Precursor

**Fig. 1:**
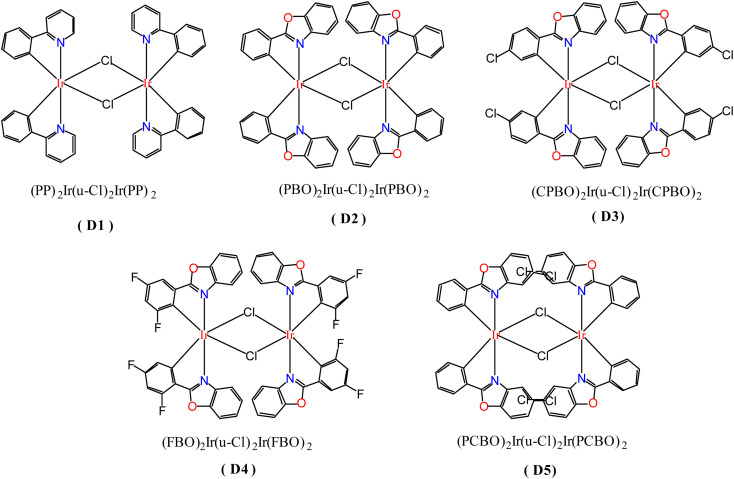
Molecular structures of dinuclear iridium complexes D1–D5.

**Scheme 2:**
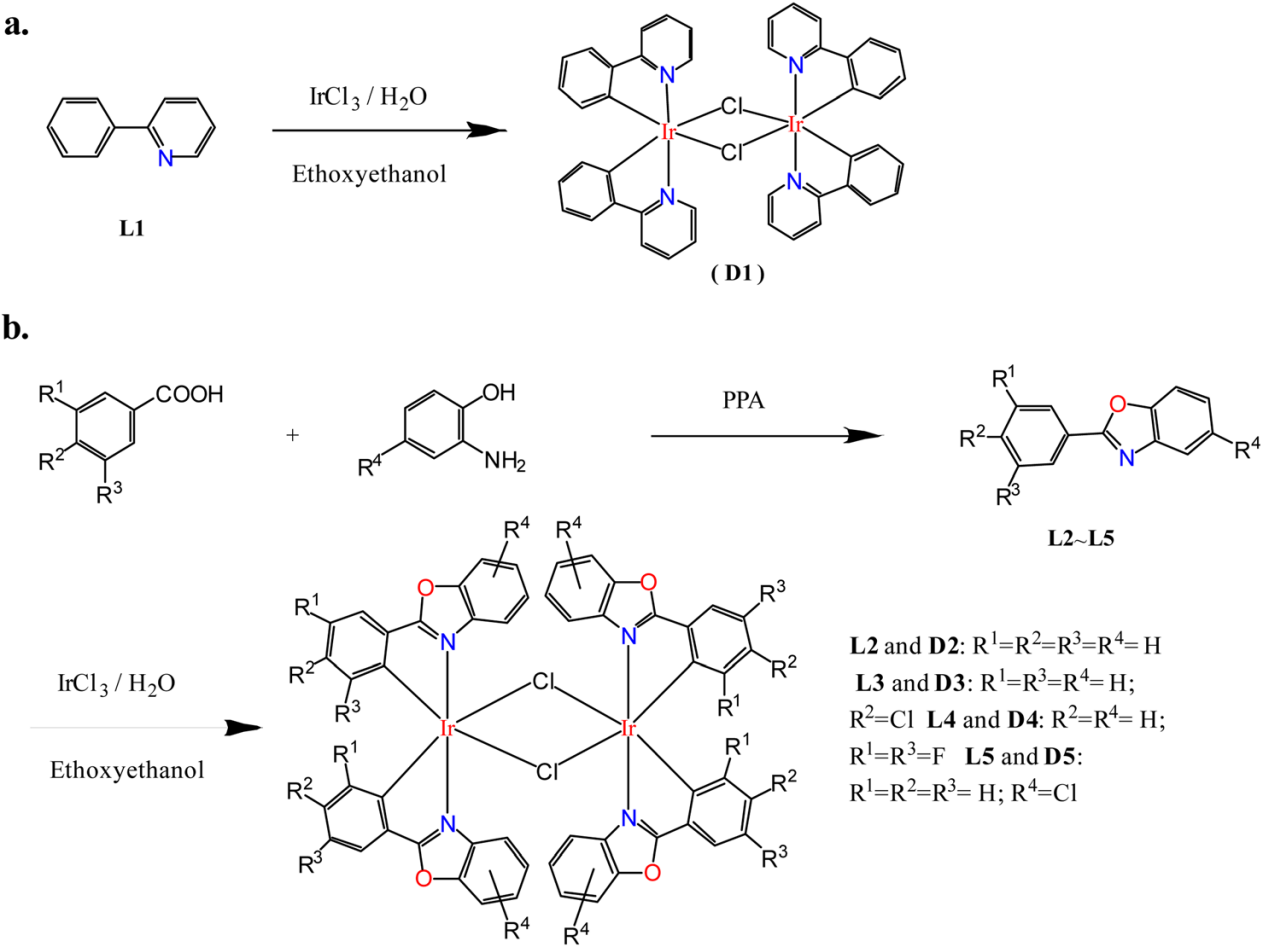
Preparation pathways of dinuclear iridium complexes D1–D5. Preparation routes for D1 (a) and D4–D5 (b).

In order to study the ligand effect of iridium complexes on catalytic alkylation for amines, dinuclear iridium precursors supported by different cyclometallated ligands with the general formula (C^N)_2_Ir(μ-Cl)_2_Ir(C^N)_2_ (D1–D5) were prepared, as shown in Fig. 1. The dinuclear iridium complex (pp)_2_Ir(μ-Cl)_2_Ir(pp)_2_, pp = 2-phenylpyridyl, D1, was directly prepared by Nonoyama reaction of 2-phenylpyridine with iridium chloride (Scheme 2a),^32,33^ whereas, (pbo)_2_Ir(μ-Cl)_2_Ir(pbo)_2_ (D2), (cpbo)_2_Ir(μ-Cl)_2_Ir(cpbo)_2_ (D3), (fpbo)_2_Ir(μ-Cl)_2_Ir(fpbo)_2_ (D4), and (pcbo)_2_Ir(μ-Cl)_2_Ir(pcbo)_2_(D5) (Scheme 2b), were prepared by the reactions of iridium chloride with a series of benzoxazole derivative ligands including 2-phenylbenzoxazole (pbo) (L2), 2-(4-chlorophenyl)benzoxazole (cpbo) (L3), 2-(3.5-difluorophenyl)benzoxazole (fpbo) (L4) and 2-phenyl-5-chlorobenzoxazole (pcbo) (L5), which were obtained by the Philips' condensation method.^34^ All the ligands and chloro-bridged dimers were isolated and characterized by ^1^H NMR, ^13^C NMR, FAB-MS spectrometry, and elemental analyses.

### Initial catalytic studies

#### Initial study on the catalytic ability of the catalytic system

**Scheme 3:**
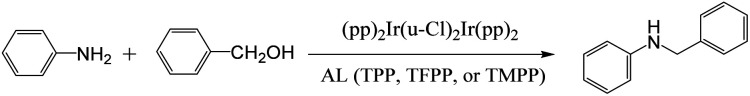
The catalytic system using D1 as the precursor and triphenylphosphine as the auxiliary ligand forms C–N bonds.

To evaluate the catalytic ability of dinuclear iridium complexes in C–N bond formation catalytic systems, we used triphenylphosphine derivatives, including triphenylphosphine (TPP), tris(4-methoxyphenyl)phosphine (TMPP) and tris(4-fluorophenyl)phosphine (TFPP) as auxiliary ligands (ALs) to open the Ir_2_Cl_2_ metallocycle of D1, creating the catalytic site for the *N*-alkylation of aniline using benzyl alcohol as the alkylating agent (Scheme 3).

**Fig. 2:**
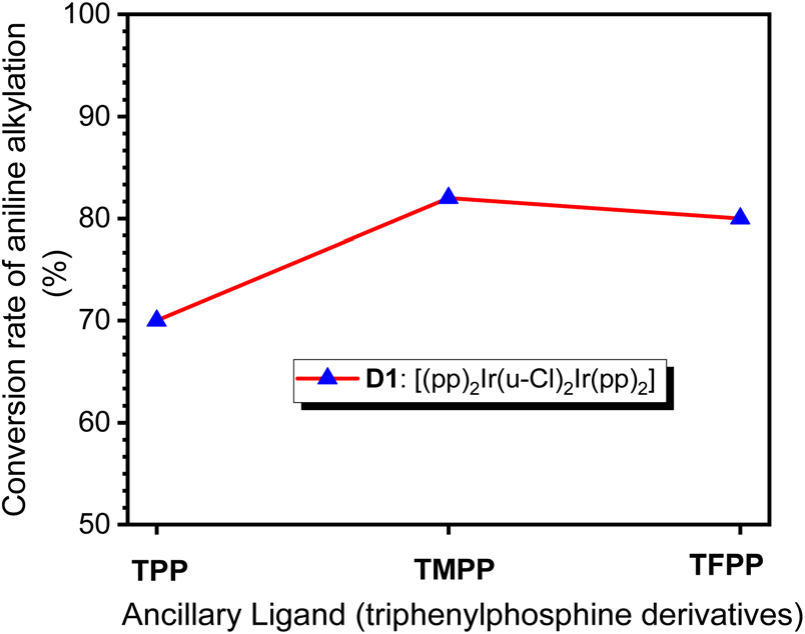
Conversion of aniline into *N*-benzylaniline in catalytic systems using D1 as precursor and TPP, TMPP or TFPP as auxiliary ligand.

When TPP, TMPP and TFPP were used as auxiliary ligands, the conversions of aniline to *N*-benzylaniline were 70, 82 and 80%, respectively (Fig. 2), suggesting that the nature of the auxiliary ligand affects the performance of C–N bond formation in amine *N*-alkylation.

**Fig. 3:**
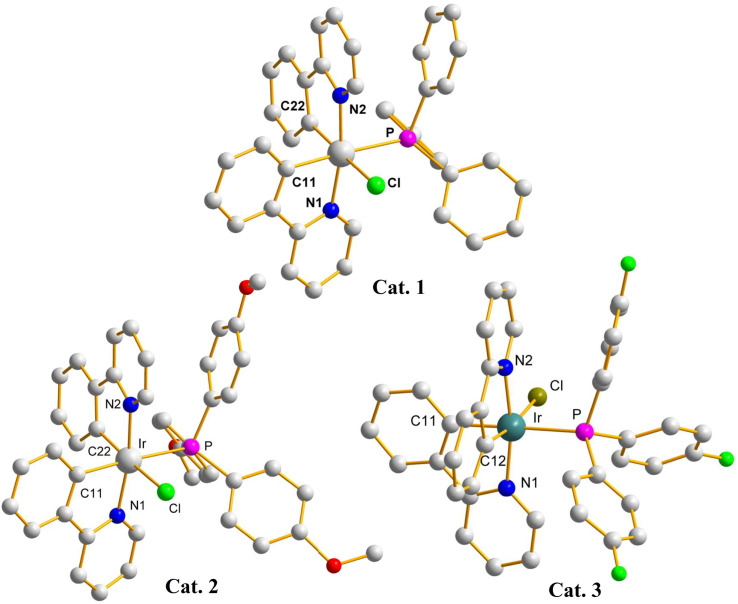
X-ray crystal structures of Cat. 1, Cat. 2, and Cat. 3.

The use of different ALs that results in different conversions may indicate the formation different iridium catalysts upon reactions. Catalysts of these reactions have been isolated and characterized by using single-crystal X-ray crystallography (crystal data for Cat. 1–Cat. 3 are listed in Table S1†). Fig. 3 depicts the crystal structures of (pp)_2_Ir(Cl) (TPP), Cat. 1, (pp)_2_Ir(Cl) (TMPP), Cat. 2, and (pp)_2_Ir(Cl)(TFPP), Cat. 3. The two nitrogen atoms are trans to each other while the phosphorus and chlorine atoms are cis to each other, resulting in the distorted octahedral geometries for the Ir(iii) metal centers. Selected bond distances and angles for Cat. 1–Cat. 3 are listed in Table S2,† showing that the Ir–P bond length of Cat. 2 [2.4388(16) Å] is longer than those of Cat. 1 [2.4074(6) Å] and Cat.3 [2.419(3) Å]. The steric effect of TMPP is thus stronger than those of TPP and TFPP, resulting in longer Ir–P bond in Cat. 2 and weaker bond strength between the central metal and TMPP. Cat. 2 is thus much easier to release TMPP ligands to provide active sites for the incoming reagents.

**Fig. 4:**
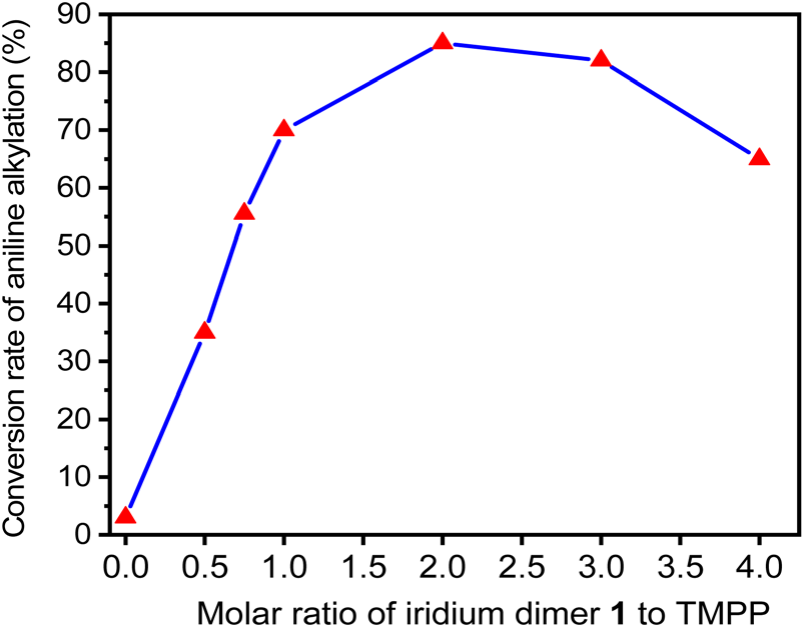
Conversion of aniline into *N*-benzylaniline in D1/TMPP catalytic systems with different proportions of D1 and TMPP.

Therefore, TMPP was chosen to evaluate the effect of bidentate ligands (C^N) on C–N bond formation in various dinuclear iridium complexes. First, we performed a series of catalytic reactions by using different ratios of D1 and TMPP to optimize the catalytic conditions. The molar ratio of D1, aniline and benzyl alcohol are 0.015 : 1 : 2, while the molar ratio of TMPP to D1 ranges from 0 to 4. The reactants were placed in Schlenk tubes, reacted at 160 °C for 24 hours, and the composition of the reaction mixture was determined by GC-MS. Fig. 4. Shows that *N*-alkylated products are rarely observed under reaction conditions without TMPP. The conversion of aniline to *N*-benzylaniline increases with increasing the molar ratio of TMPP to D1 and reaches a maximum when the molar ratio is 2. When the molar ratio of TMPP to D1 was greater than 2, the conversion gradually decreases. This suggests that the fully equivalent auxiliary ligand TMPP is necessary to open the bridging chlorine structure, but excess auxiliary ligands are detrimental to the reaction because they inhibit the release of TMPP and reduce the density of catalytic species.

**Fig. 5:**
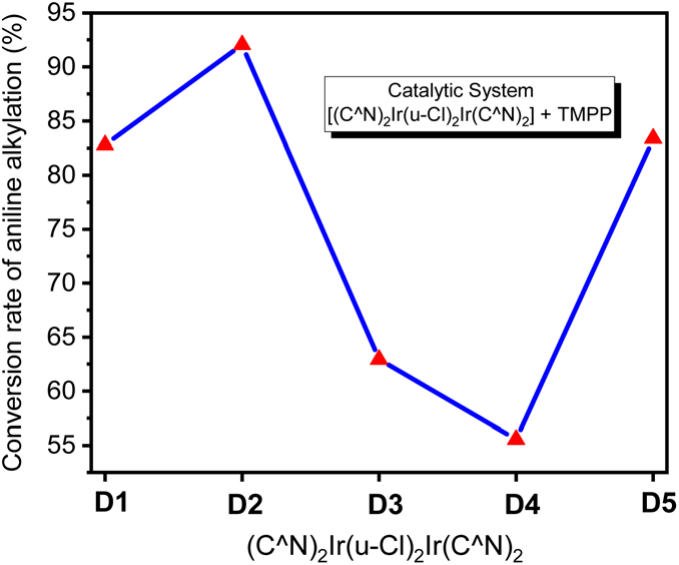
Conversion of aniline into *N*-benzylaniline in D1/TMPP, D2/TMPP, D3/TMPP, D4/TMPP, and D5/TMPP catalytic systems.

Based on optimized conditions, the dinuclear iridium complexes D1–D5 were applied to the *N*-alkylation of aniline. Fig. 5 shows that D2 with the C^N ligand phenylbenzoxazole has better performance in C–N bond formation than D1 with the phenylpyridyl group. However, the electron-withdrawing groups on the C^N ligand of D3, D4, and D5 greatly weakened the performance of C–N bond formation, and a cumulative effect was also observed for D4.

**Fig. 6:**
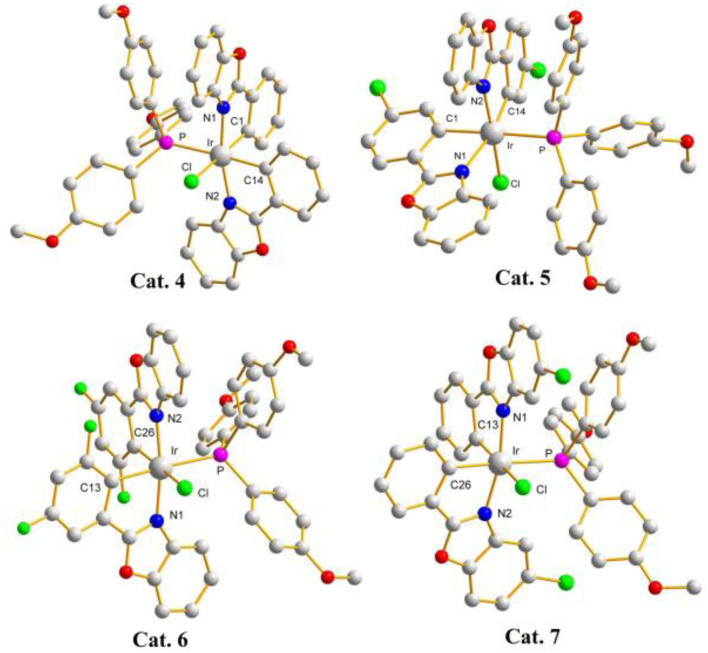
X-Ray crystal structures of Cat. 4, Cat. 5, Cat. 6, and Cat. 7.

**Fig. 7:**
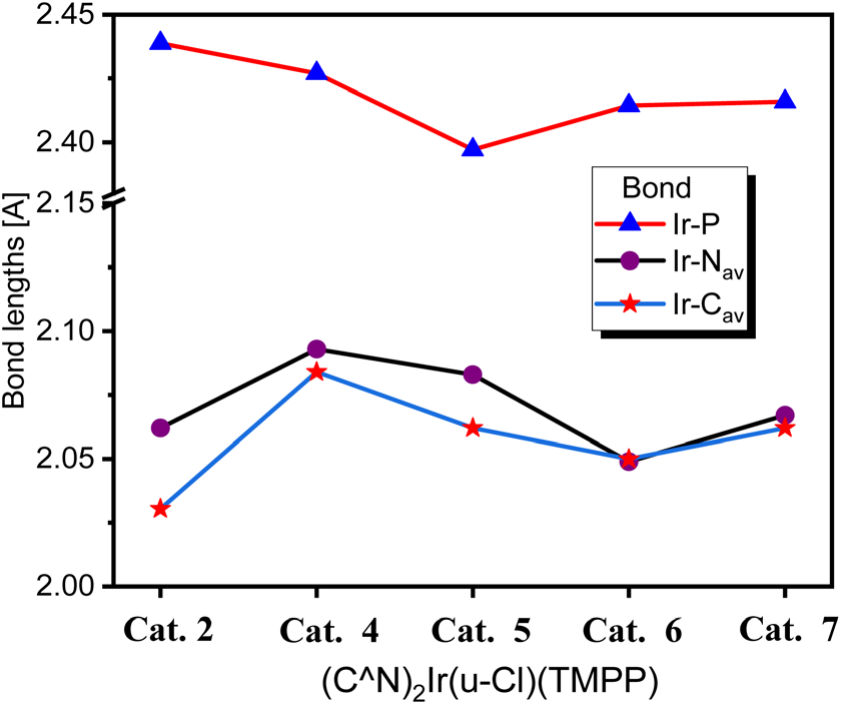
Comparison of bond distances between iridium and coordinating atoms in Cat. 2, Cat. 4, Cat. 5, Cat. 6 and Cat. 7.

Catalysts of these reactions have also been isolated and characterized by X-ray crystallography (crystal data for Cat. 4–Cat. 7 are listed in Tables S3– S4†). Single-crystal structures of catalysts (pbo)_2_Ir(Cl) (TMPP), Cat. 4, (cpbo)_2_Ir(Cl) (TMPP), Cat. 5, (fpbo)_2_Ir(Cl) (TMPP), Cat. 6, and (pcbo)_2_Ir(Cl)(TMPP), Cat. 7, are represented with ORTEP diagrams in Fig. 6. All catalysts adopt twisted octahedrons, with *cis*-CC, *trans*-N–N and *cis*-P–Cl arrangements. Selected bond distances and angles of Cat. 4–Cat. 7 are listed in Table S5,† and the comparison of bond distances between iridium and coordination atoms is shown in Fig. 7.

Electronegative groups (such as fluorine and chlorine) bonded to the phenylbenzoxazole enhance the bonding of the catalysts, resulting in shorter bond distances between iridium and coordinating atoms. For catalysts composed of phenylbenzoxazole derivatives as C^N ligands, Cat. 4 has the longest Ir–P, Ir–N_AV_ and Ir–C_AV_ bond lengths, which are 2.427 (4), 2.093 (6) and 2.067(9) Å, respectively. As mentioned above, the catalytic system with D2 as precursor and TMPP as auxiliary ligand exhibited the best C–N bond formation performance in the *N*-alkylation of amines, indicating that the catalytic system has two favourable properties for C–N bond formation. One is that the bond strength between iridium and the phosphorus atom of TMPP is stronger than that between iridium and coordinated chlorine in chloro-bridged dimers (C^N)_2_Ir(μ-Cl)_2_Ir(C^N)_2_, so TMPP can effectively open the dimer structures, and for D2, the bond strength between iridium and the phosphorus of TMPP is suitable for releasing TMPP to create catalytic sites. As the bond strength between iridium and phosphorus atoms in TMPP increases, the release of TMPP becomes difficult and the catalytic activity of C–N bond formation decreases. The second effect is that the steric effect of Cat. 4 is less hinder than catalysts with electronegative groups on the phenyl or benzoxazole groups. As shown in Fig. 7, when the electronegative group is located on the he C^N ligand, the bonding strength between iridium and coordination atom is enhanced, shortening the bond lengths of Ir–P, Ir–N_AV_ and Ir–C_AV_, leading to a centrally constricted structure and raising the barrier to C–N bond formation. The Ir–P bond length of Cat. 2 is longer than that of Cat. 4, so in principal Cat. 2 should release TMPP more easily than Cat. 4, and then Cat. 2 should have a higher catalytic activity for C–N bond formation than Cat. 4. Upon further detailed inspection, the bond lengths of Ir–N_AV_ and Ir–C_AV_ of Cat. 2 are significantly shorter than those of Cat. 4, suggesting that the steric effect of Cat. 2 is more hinder than Cat. 4. Combining of the two factors affecting catalytic activity, catalytic system using D2 as a precursor and TMPP as an auxiliary ligand showed better performance of C–N bond formation than that using D1 and TMPP. In addition, the Ir–P bond length of Cat. 5 is shorter than that of Cat. 6, but the Ir–N_AV_ and Ir–C_AV_ bond lengths of Cat. 5 are significantly longer than those of Cat. 6. This again shows that even if the catalytic species is easily formed, the steric effect of the catalyst is an important factor.

**Fig. 8:**
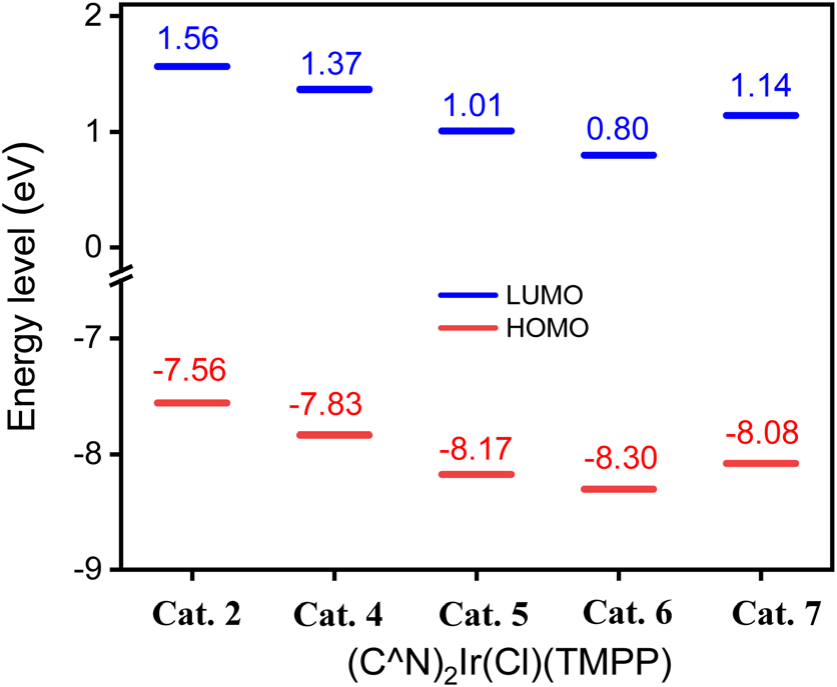
HOMO and LUMO energy levels of t Cat. 2, Cat. 4, Cat. 5, Cat. 6 and Cat. 7.

**Fig. 9:**
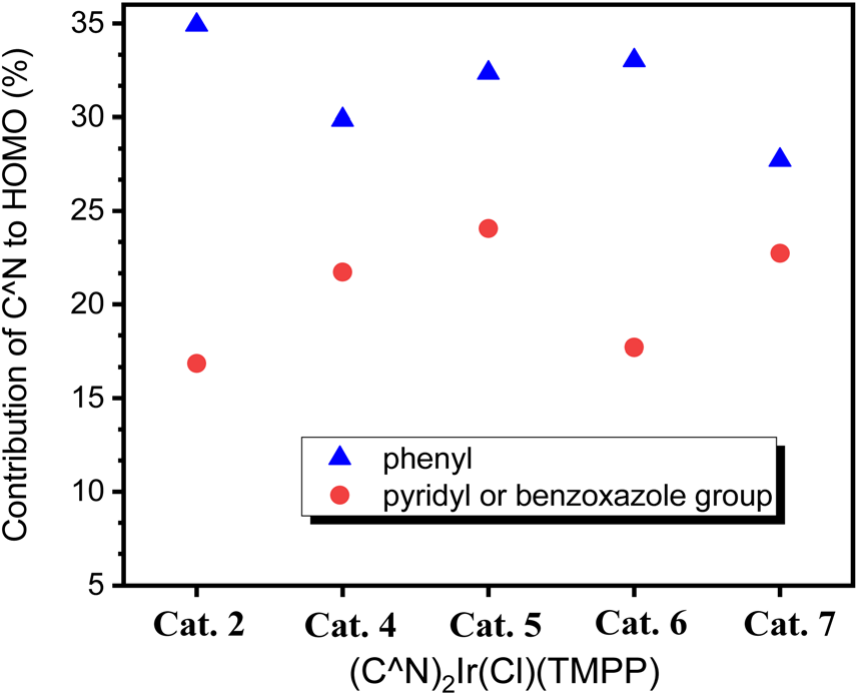
HOMO contribution of C^N ligands for Cat. 2 and Cat. 4–Cat. 7.

To further investigate the effect of the ligand C^N on the molecular structures, calculation based on DFT (B3LYP/LANL2DZ level) for Cat. 2 and Cat. 4–Cat. 7 were performed. The highest occupied molecular orbital (HOMO) energy levels of Cat. 2 and Cat. 4–Cat. 7 are 1.56, 1.37, 1.01, 0.80 and 1.14 eV, respectively. The energy levels of their lowest unoccupied orbital (LUMO) are −7.56, −7.83, −8.17, −8.30 and −8.08 eV, respectively. Fig. 8 shows that the electron-withdrawing group bonded on the phenylbenzoxazole can stabilize the HOMO and LUMO of the catalyst, thereby inhibiting the release of the coordination ligand. Among them, the MO energy level of Cat. 5 having an electron-withdrawing group bonded to the phenyl group of the C^N ligand is lower than that of Cat. 7 having an electron-withdrawing group bonded to the benzoxazole group of C^N ligand. The phenyl groups of C^N ligands contribute 34.92, 29.86, 32.35, 33.02 and 27.72% to the HOMOs of Cat. 2 and Cat. 4–Cat. 7, respectively. The pyridyl or benzoxazole groups of C^N ligands contribute 16.86, 21.72, 24.05, 17.71 and 22.73% to the HOMO of Cat. 2 and Cat. 4–Cat. 7, respectively. Fig. 9 shows that the phenyl group of the C^N ligand is the main contributor to the HOMO; therefore, catalysts with electron-withdrawing groups bonded to the phenyl groups of C^N ligands have a greater impact on the energy levels of HOMOs.

**Fig. 10:**
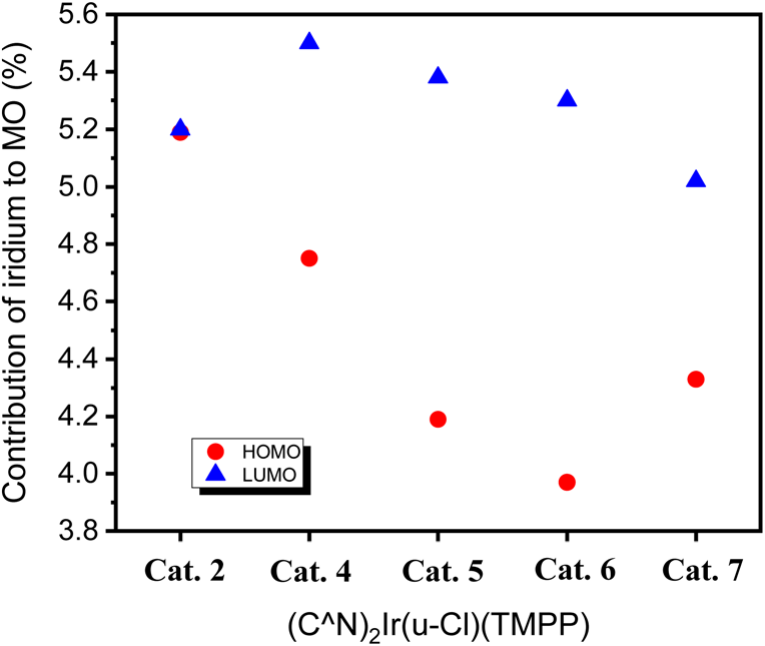
HOMO and LUMO contribution of iridium to Cat. 2 and Cat. 4–Cat. 7.

Since the iridium atom is the catalytic centre of the complex, the contribution of iridium to the frontier orbitals was also investigated. The contribution of iridium to the HOMOs of Cat. 2 and Cat. 4–Cat. 7 are 5.19, 4.75, 4.19, 3.97 and 3.33%, respectively. The contribution of iridium to the LUMOs of Cat. 2 and Cat. 4–Cat. 7 are 5.19, 5.50, 5.48, 5.30 and 3.02%, respectively. Fig. 10 shows that the LUMO of Cat. 4 has the largest proportion of iridium among all catalysts, and the HOMO of Cat. 4 has the largest proportion of iridium among the phenylbenzoxazole derivatives. This is similar to the trend of C–N bond formation in the above catalytic system, implying a positive correlation between the contribution of iridium to the frontier orbitals and the activity of the catalytic species.

**Fig. 11:**
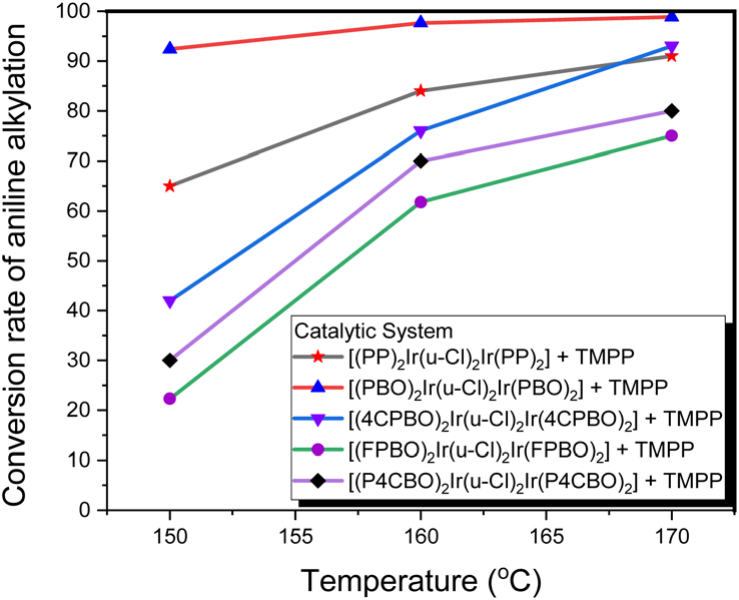
Conversion of aniline into *N*-benzylaniline in D1/TMPP, D2/TMPP, D3/TMPP, D4/TMPP, and D5/TMPP catalytic systems at different reaction temperatures.

Based on the above information, we can infer that the stability of the bridging chlorine structures of the dinuclear iridium precursors (D1–D5) should be different from each other, which exhibit the characteristic of C–N bond formation activities that are dependent on the temperature. Fig. 11 shows that, for the catalytic system D4/TMPP, only about 20% conversion was observed at 150 °C, whereas, a moderate yield (about 55%) was observed at 160 °C, and only about 65% was reached at a rather high temperature of 170 °C. This indicates that the bridging chlorine structure of precursor D4 is quite inert and difficult to be opened by TMPP. The performance of the D3/TMPP catalytic system was better than that of D4/TMPP, and the highest yield was 70%. The performance of the D5/TMPP catalytic system was also significantly temperature-dependent, but the conversion was as high as 88%. The maximum conversion rate of the D1/TMPP catalytic system (85%) was slightly lower than that of the D5/TMPP catalytic system, but at 150 °C the conversion rate of the D1/TMPP catalytic system (65%) was much higher than that of the D5/TMPP catalytic system (42%). Importantly, the D2/TMPP catalytic system exhibited stable and excellent performance on C–N bond formation, with conversions of 92% at 150 °C, 98% at 160 °C, and 100% at 170 °C.

While the D1/TMPP and D2/TMPP catalytic systems provided moderate to excellent yields for the *N*-alkylation of amines at 150 °C, the yields for the other catalytic systems were all below 50%. When the reaction temperature was increased from 150 °C to 160 °C, the yields of all catalytic systems increased dramatically. When the reaction temperature increased from 160 °C to 170 °C, the productivity of each catalytic system still increased, but the increase was much smaller than when the reaction temperature increased from 150 °C to 160 °C. Also, some catalytic systems form by-products at 170 °C. Therefore, in the following study, the reaction at 160 °C was used to evaluate the performance of the catalytic systems for C–N bond formation.

**Fig. 12:**
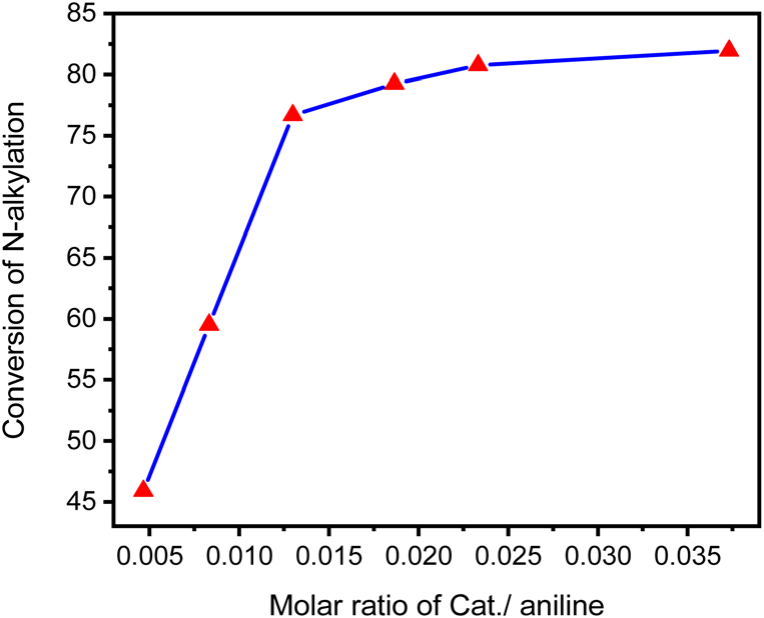
Conversion of aniline into *N*-benzylaniline in the D1/TMPP catalytic system based on different loading ratios of precursor D1 and auxiliary ligand TMPP.

A series of D1/TMPP catalytic reactions based on different catalyst loading ratios were carried out to evaluate the conversion of amines to *N*-alkylated products, where the molar ratio of precursor D1 to auxiliary ligand TMPP was fixed at 1 : 2, the molar ratio of precursor D1 to amine is in the range of 0.0047 to 0.0373 (mmol mmol^−1^), and the molar ratio of amine to alcohol was fixed at 1 : 2. Reactions were carried out in Schlenk tubes at 160 °C for 24 h, and the composition of the reaction mixture was determined by GC-MS and summarized in Fig. 12. At low catalyst loading (0.0047 mmol mmol^−1^), a low concentration of the alkylated product (*N*-dibenzylaniline) (45.9%) was detected. When the catalyst loading ratio increased to 0.0123 mmol mmol^−1^, the conversion of amine alkylation products increased sharply (up to 76.7%). When the catalyst loading was 0.0187 mmol mmol^−1^, the amine conversion increased slightly to 79.25%. When the catalyst loading was further increased, the amine conversion did not increase significantly, even if the catalyst loading was 0.0373 mmol mmol^−1^, the amine conversion only increased to 81.95. Therefore, we adopted a catalyst loading ratio of 0.015 mmol mmol^−1^ as a standard for evaluating other catalytic reactions.

**Fig. 13:**
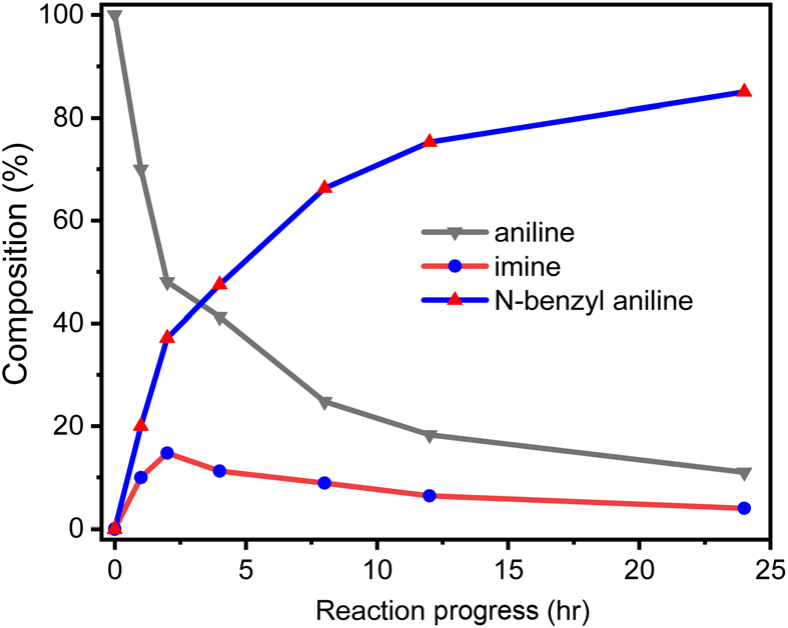
Reaction progress of *N*-alkylation of aniline.

We monitored the composition change with the reaction time when aniline and benzyl alcohol were reacted in the D1/TMPP catalytic system, the catalyst loading ratio was fixed at 0.015 mmol mmol^−1^, and the composition of the reaction mixture was determined by GC-MS. Fig. 13 shows that at the beginning of the reaction (the first 30 minutes), the aniline disappeared significantly, the concentration of the product (*N*-benzylaniline) increased rapidly, and the imine was gradually formed. Thereafter the aniline was still consumed at a moderate rate and the concentration of the product (*N*-benzylaniline) increased steadily but the concentration of the imine decreased gradually. It shows that at this stage, the conversion rate of imine to *N*-benzylaniline is faster than that of aniline to imine. After a reaction time of 12 hours, the decrease of aniline and imine slowed down, and the increase of *N*-benzylaniline also slowed down. After 24 hours of reaction, the concentrations of aniline, imine and *N*-benzylaniline did not change significantly. Therefore, in order to compare the capabilities of the catalytic systems, the time of the catalytic reaction was set as 24 hours. Except for aniline, benzyl alcohol, imine and *N*-benzyl aniline, no other substances were detected in the reaction system. In addition, no benzaldehyde was detected in the reaction system containing only the catalyst and benzyl alcohol without aniline, implying that the imine should be released from an intermediate in the catalytic cycle rather than through the reaction of the amine with the aldehyde produced by the reaction of the catalyst with benzyl alcohol.

#### Substrate scope

Based on optimized conditions, we used three catalytic systems, D1/TMPP, D2/TMPP, and D4/TMPP, to study the substrate scope of *N*-alkylation of amines in the hydrogen borrowing reaction. The conversion of amines to *N*-alkylated products (P1–P15) is summarized in Table 1 showing that all catalytic systems have good performance in terms of C–N bond formation, for example, the conversion of aniline to *N*-benzylaniline using D1/TMPP, D2/TMPP and D4/TMPP catalyst systems were 85, 98 and 65% respectively. Substituted alcohols such as 4-chlorobenzyl alcohol and 4-methoxybenzyl alcohol can also alkylate aniline well to obtain the corresponding secondary amines P2 and P3, and the conversion rate is good in the D1/TMPP catalytic system (average 84%), excellent in the D2/TMPP catalytic system (average 97%), and moderate in the D4/TMPP catalytic system (average 63%). Substituted anilines, such as *p*-methoxyaniline, 4-chloroaniline, can also be well alkylated with benzyl alcohol or substituted alcohols to give the corresponding *N*-alkylated products (P4–P9). The average conversion rate of the D1/TMPP catalytic system is 76%, the average conversion rate of the D2/TMPP catalytic system is 92%, and the average conversion rate of the D4/TMPP catalytic system is 59%. Secondary alcohols are also conducive to the alkylation of amines, resulting in corresponding *N*-alkylated products (P10–P12).

**Table 1:** *N*-Alkylation of amines in D1/TMPP, D2/TMPP and D4/TMPP catalytic systems^a^

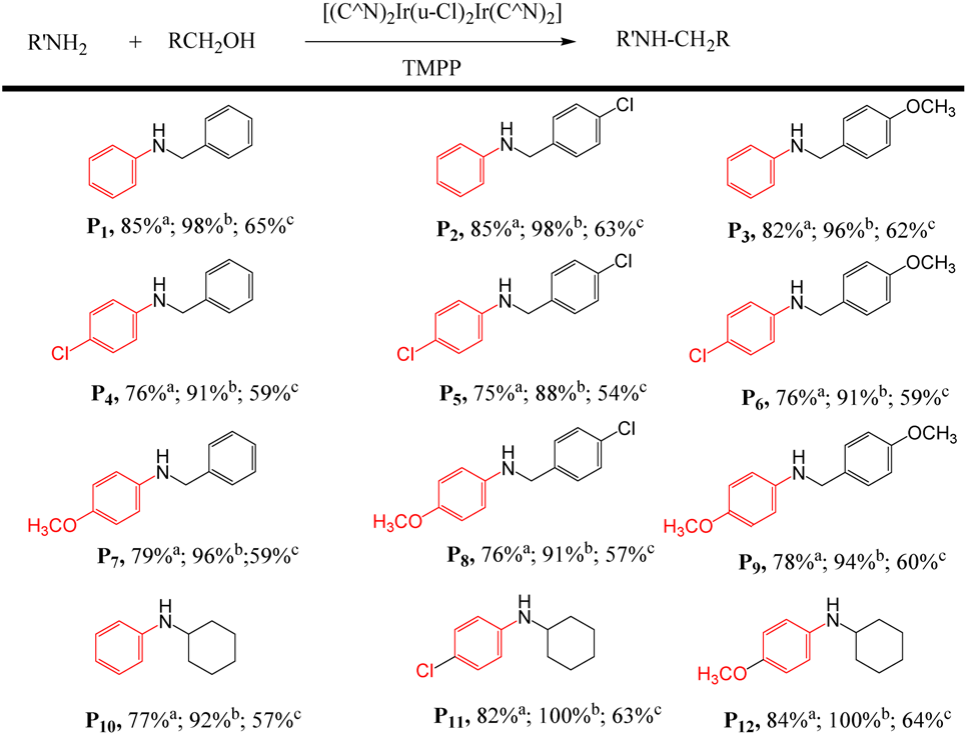

a Add a mixture of 1 mmol of amine with 2 mmol of alcohol, 0.015 mmol of dinuclear iridium complex and 0.03 mmol of TMPP into a Schlenk tube and react at 160 °C for 24 h. ^a^ Conversion in D1/TMPP catalytic system; ^b^ conversion in D2/TMPP catalytic system; ^c^ conversion in D4/TMPP catalytic system.

**Fig. 14:**
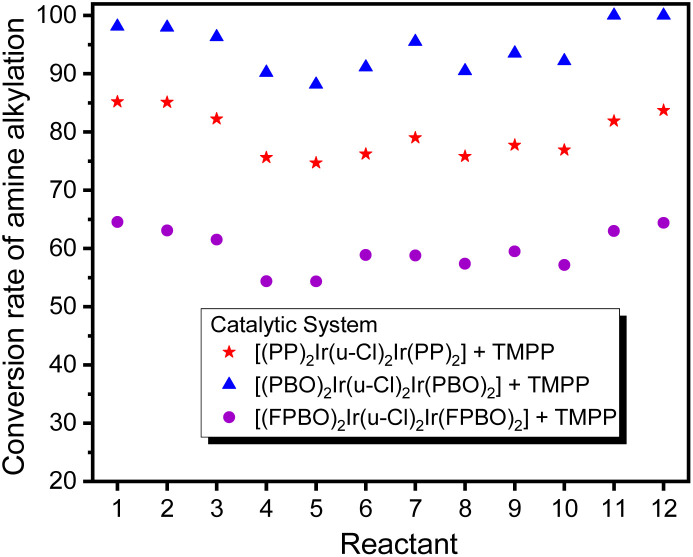
Comparison of conversion of *N*-alkylation of amines showed in Table 1.

Fig. 14 shows that in 12 C–N bond formation reactions, the D2/TMPP catalytic system exhibited excellent performance with an average conversion of about 95% and the D1/TMPP catalytic system showed good results with an average conversion of about 80%. Although the catalytic capacity of the D4/TMPP catalytic system was much lower than that of the D2/TMPP and D1/TMPP catalytic systems, it provided moderate results with an average conversion of about 55%. These indicated that all (C^N)_2_Ir(μ-Cl)_2_Ir(C^N)_2_/TMPP catalytic systems proposed here have high stability and reliability in catalyzing C–N bond formation in various substrate combinations.

#### Properties of the catalyst

Catalysts 1–7 were separated from the catalytic system and can also be directly synthesized by reacting dinuclear iridium complexes with triphenylphosphine derivatives. All catalysts are stable in air at room temperature. As a homogeneous catalyst used at low loading rates, it is difficult to accurately recover from the reaction mixture, so its recyclability was not evaluated. The turnover number (TON) and turnover frequency (TOF) of 15 catalytic systems for the conversion of aniline to *N*-benzylaniline were evaluated and summarized in Table 2. The turnover frequency of the 15 catalytic systems ranges from 2.87 to 4.17, and the turnover number ranges from 100 to 180. For catalytic systems using the same dinuclear iridium complex but different triphenylphosphine derivatives, the catalytic system using D2 showed the best performance, while for catalytic systems using the same triphenylphosphine derivatives but different dinuclear iridium complex, the catalytic system with TMPP showed the best performance.

**Table 2:** TON and TOF of catalytic system^a^

Iridium complex	Catalytic system	TOF (s^−1^) (× 10^−3^)	TON
D1 [(pp)_2_Ir(μ-Cl)]	D1/TPP	3.01	130
	D1/TMPP	3.47	150
	D1/TFPP	3.24	140
D2 [(pbo)_2_Ir(μ-Cl)]	D2/TPP	3.94	170
	D2/TMPP	4.17	180
	D2/TFPP	4.07	176
D3 [(cpbo)_2_Ir(μ-Cl)]	D3/TPP	3.15	136
	D3/TMPP	3.70	160
	D3/TFPP	2.87	124
D4 [(fpbo)_2_Ir(μ-Cl)]	D4/TPP	2.31	100
	D4/TMPP	3.01	130
	D4/TFPP	2.78	120
D5 [(pcbo)_2_Ir(μ-Cl)]	D5/TPP	3.24	140
	D5/TMPP	3.80	164
	D5/TFPP	3.01	130

a Add a mixture of 1 mmol of amine with 2 mmol of alcohol, 0.005 mmol of dinuclear iridium complex and 0.01 mmol of triphenylphosphine derivatives into a Schlenk tube and react at 160 °C for 12 h. The composition of the reaction mixture was determined by GC-MS.

#### Reaction mechanism exploration

In order to gain a deeper understanding of the *N*-alkylation process, we performed some reactions to study the impact of the environment of the catalysts on the *N*-alkylation process.

**Fig. 15:**
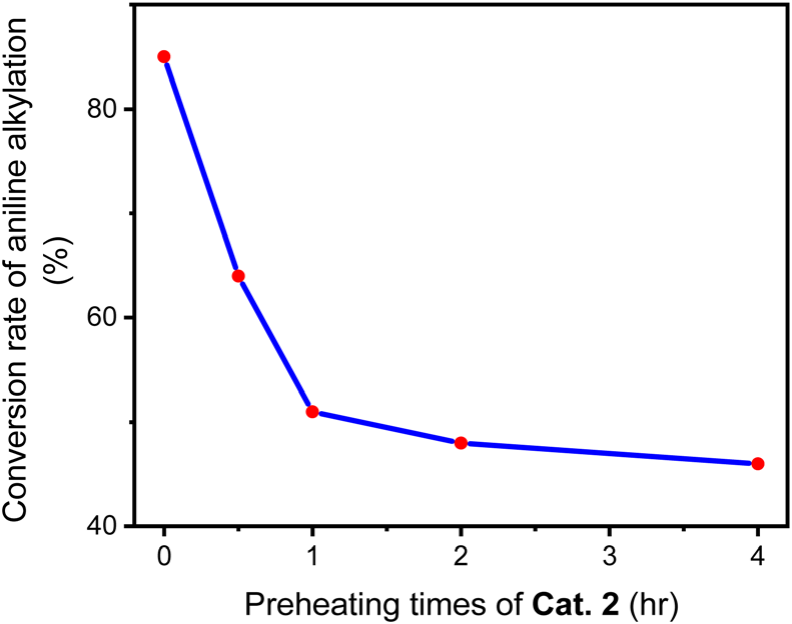
Conversion of aniline into *N*-benzylaniline by catalyst Cat. 2 pre-treated for different times.

**Scheme 4:**
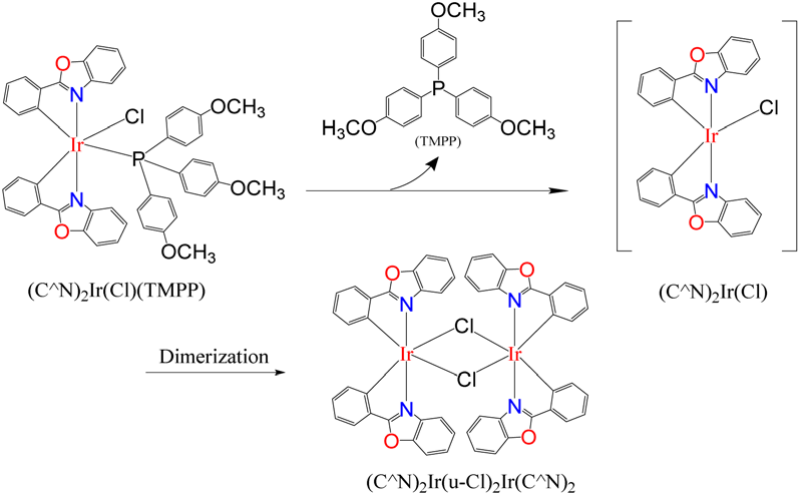
Cat. 2 releases the TMPP ligand to reform the dinuclear iridium complex D1.

**Fig. 16:**
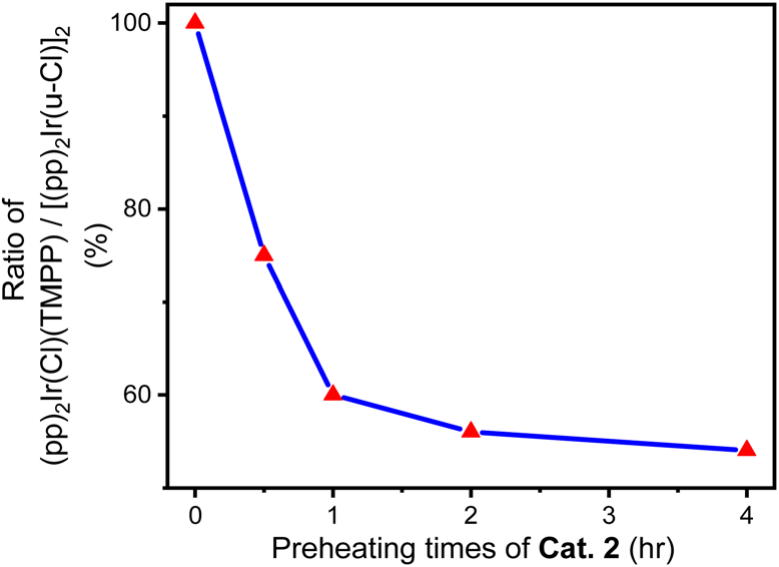
Relationship between the ratio of (pp)_2_Ir(Cl)(TMPP)/[(pp)_2_Ir(μ-Cl)]_2_ and the preheating time of Cat. 2.

In a Schlenk tube, 0.030 mmol Cat. 2 was mixed with 5 mmol toluene and heated at 120 °C for some time under nitrogen atmosphere. After evaporating toluene, add 1 mmol aniline and 2 mmol benzyl alcohol to the reaction tube, tighten and heat at 160 °C for 24 hours. A series of reactions based on different preheating times of Cat. 2 were carried out to evaluate the relationship between Cat. 2 preheating time and C–N bond forming ability. Fig. 15 shows that the conversion of aniline to *N*-benzylaniline in the reaction using Cat. 2 as catalyst without preheating is similar to the reaction using the D1/TMPP system (85%). As the preheating time of Cat. 2 increases, the conversion rate of aniline into *N*-benzylaniline decreases rapidly. The conversion rates of aniline to *N*-benzylaniline in experiments with 0.5, 1, 2 and 4 hours of preheating were 64, 51, 48 and 46% respectively, indicating that during preheating in a nitrogen atmosphere, Cat. 2 will dissociate and release the TMPP ligand to re-form the dinuclear iridium complex [(pp)_2_Ir(μ-Cl)]_2_ (D1) (Scheme 4), resulting in a decrease in the conversion rate of aniline. Fig. 16 shows that the ratio of (pp)_2_Ir(Cl)(TMPP)/[(pp)_2_Ir(μ-Cl)]_2_ decreases with increasing Cat. 2 preheating time (monitored by NMR).

**Fig. 17:**
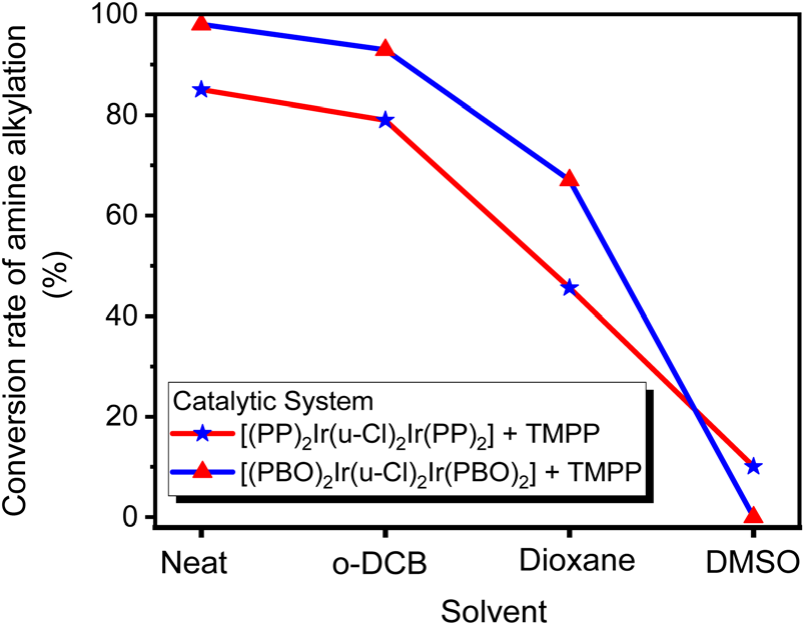
Effect of solvents on the performance of catalytic systems D1/TMPP and D2/TMPP.

**Scheme 5:**
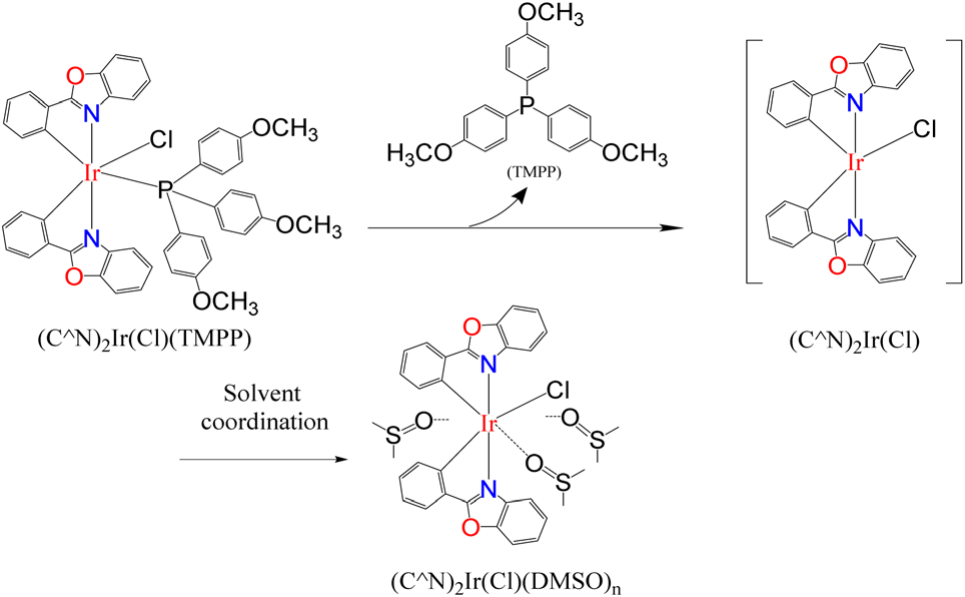
Inhibition of Lewis donor solvents on catalytic system performance.

In most cases, solvents are used to facilitate chemical reactions, but sometimes, instead of promoting the reaction, they hinder it. The catalytic reaction systems mentioned above are extremely sensitive to solvents. Three solvents, *o*-dichlorobenzene (*O*-DCB), dioxane and dimethyl sulfoxide (DMSO), were added to two catalytic systems D1/TMPP and D2/TMPP to study the effect of solvents on the performance of the catalytic system. In a Schlenk tube, 0.015 mmol of dinuclear iridium complex D1 or D2 was mixed with 0.030 mmol of TMPP, 2 mmol of solvent (*O*-DCB, dioxane, or DMSO), 1 mmol of aniline, and 2 mmol of benzyl alcohol. The reaction was carried out at 160 °C for 24 hours, and the composition of the reaction mixture was determined by GC-MS. The C–N bond formation activity is suppressed about 5% in *O*-DCB, 30–40% in dioxane, and 80∼100% in DMSO (Fig. 17), showing that aprotic solvents hinder the catalytic system. Furthermore, an aprotic solvent with a higher dipole moment (DMSO, *μ* = 3.96) inhibits the conversion of amine alkylation more severely than an aprotic solvent with a lower dipole moment (dioxane, *μ* = 2.55). This shows that better solvating solvents significantly reduce the reactivity of the catalyst and that the Lewis donor suppresses the activity of the catalytic species (Scheme 5).

**Scheme 6:**
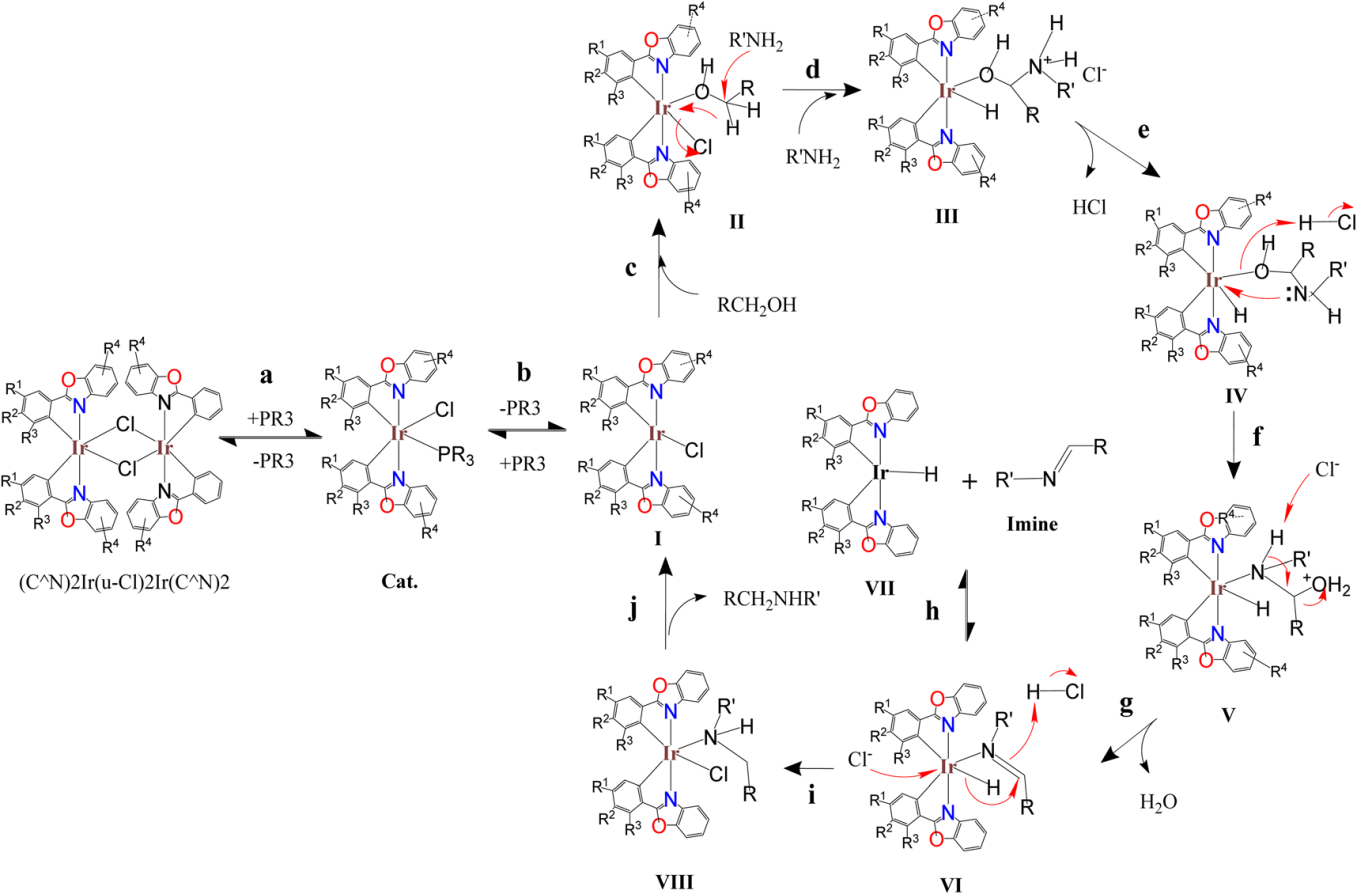
Proposed mechanism for the catalytic C–N bond formation.

The above studies show that in the catalytic system, the Ir_2_Cl_2_ metal ring of the iridium dimer should be opened early to form an iridium monomer catalyst. The iridium monomer can release auxiliary ligands and produce coordination unsaturated species for catalysis, but can also recombine to form dinuclear iridium complex. Imines are observed in the catalytic system and are released from an intermediate in the catalytic cycle rather than through the reaction of the amine with the aldehyde produced by the reaction of the catalyst with benzyl alcohol. The vacancies of the coordination unsaturated compound are often coordinated with the lone pair of electrons of the oxygen atoms of dioxane and DMSO, and can also be coordinated with the lone pair of electrons of the alcohol to form an alcohol coordination intermediate. Based on the above information and preliminary mechanistic studies,^35–37^ a plausible mechanism for C–N bond formation in the (C^N)_2_Ir(μ-Cl)_2_Ir(C^N)_2_/AL catalytic systems was proposed (Scheme 6). In the first step of the catalytic cycle (step a), the bridge chlorine structure ((C^N)2Ir(μ-Cl)2Ir(C^N)2) of the precursor is opened by the attack of the auxiliary ligand (AL) to form a catalyst. The catalyst releases the auxiliary ligand to form coordinatively unsaturated species I (step b). The alcohol binds to the vacant coordination site of I to form the alcohol-coordinated intermediate II (step c). The amine attacks the carbon of the alcohol coordinated to the metal, causing the hydride to transfer from the carbon to the metal, releasing chloride ions, forming the ammonium hydride iridium complex III (step d). Hydrogen chloride is released to form iridium hydride complex IV (step e). After oxygen protonation, the lone pair electron of nitrogen bonds to the metal, displacing the metal–oxygen bond to form the amine hydride iridium complex V (step f). Dehydration of V to form imine hydride iridium complex VI (step g). The imine-iridium hydride complex VI undergoes two reactions. One is to release the imine in a reversible reaction to form the iridium hydride complex VII (step h); the second pathway is to transfer the hydride from the metal to the carbon of the imino group to form the amine chloride iridium complex VIII (step i). Finally, the product is released from complex VIII and regenerates the coordinated unsaturated species I (step j).

#### Synthetic applications

N-Heterocycles are important structures in biochemical and pharmaceutical compounds, usually constructed through many complex steps. Below, we use the D2/TMPP catalytic system to construct heterocycles in a more compact manner.

First, the D2/TMPP catalytic system can use simple cyclic amines to construct complex target molecules with high yields in the cleaning process. For example, aniline, 4-chloroaniline and 4-methoxyaniline reacted with pyrrolidine to form benzylpyrrolidine derivatives P13, P14 and P15 with yields of 91%, 87% and 85% respectively (Table 3). *N*-Benzylpyrrolidine derivatives have undergone extensive biological evaluation in the treatment of Alzheimer's disease (AD) for ameliorating scopolamine-induced amnesia and amyloid beta-induced cognitive dysfunction. Such derivatives are also used to reduce brain AChE activity, antioxidant potential and balance enzyme inhibition of cholinesterase. In addition, piperazine was reacted with aniline to form benzylpiperazine (BZP) P16, a recreational drug with euphoric properties, in 94% yield. Piperazine reacts with 4-chloroaniline to form *p*-chlorobenzylpiperazine (P17) in 93% yield, which has been explored as an inhibitor that specifically binds to the 5-HT transporter (PubMed),^38^ such as [3*H*]6-nitroquinazine. Piperazine reacted with 4-methoxyaniline to produce methoxybenzylpiperazine (P18) with a yield of 95%, and its inhibitory effect on the cholinergic system was studied.

**Table 3:** Preparation of N-heterocycles in D2/TMPP catalytic systems^a^

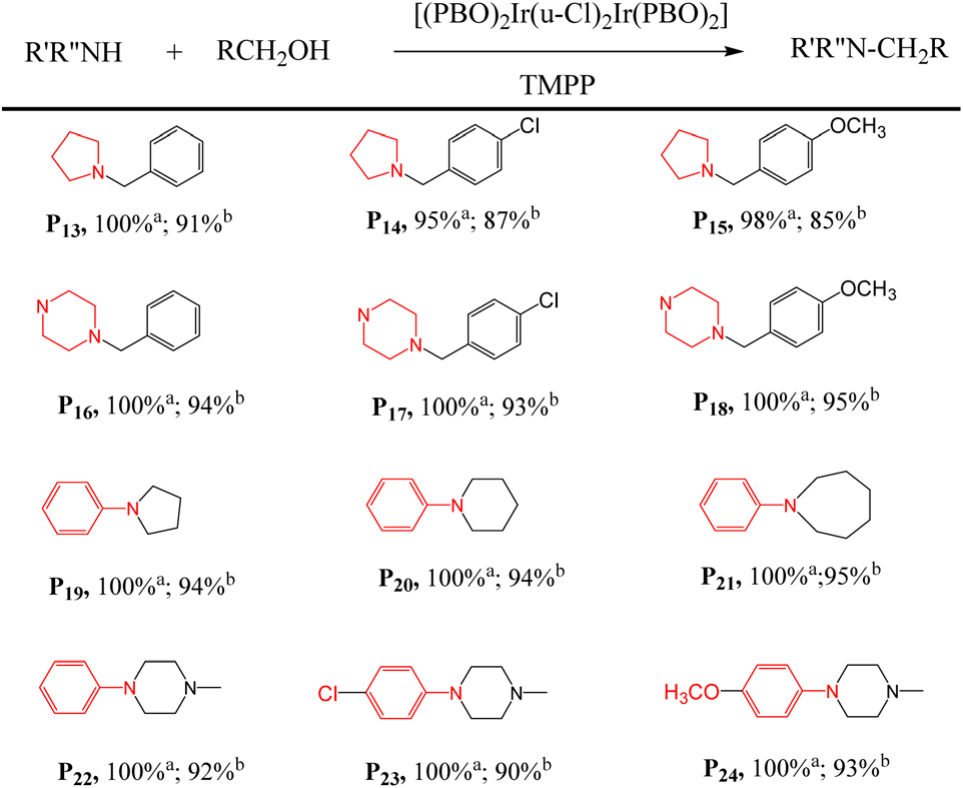

a Add a mixture of 1 mmol of amine with 2 mmol of alcohol, 0.015 mmol of dinuclear iridium complex and 0.03 mmol of TMPP into a Schlenk tube and react at 160 °C for 24 h. ^a^ Conversion in D2/TMPP catalytic system; ^b^ isolated yields.

Second, these catalytic systems can provide a cascade reaction process to construct N-heterocycles. For example, aniline and diol react through intermolecular and intramolecular reactions to form N-heterocycles, including *N*-phenylpyrrolidine (P19), *N*-phenylpiperidine (P20) and seven-membered N-heterocycle (P21). The yields were 94, 94 and 95% respectively. These nitrogen heterocycles and their derivatives are ubiquitous structural units in pharmaceuticals and fine chemicals.

Interestingly, phenylpiperazine derivatives (P22–P24) were obtained by reacting diethanolamine or *N*-substituted diethanolamine with aniline derivatives through a cascade reaction process of intermolecular and intramolecular reactions. The phenylpiperazine derivatives are characterized by the phenyl group attached to the piperazine ring. Many phenylpiperazine derivatives are medicines,^39^ such as antrafenine, bifeprunox, ciprofloxacin, dropropizine. and elopiprazole.

## Conclusions

The catalytic system composed of dinuclear iridium (C^N)_2_Ir(μ-Cl)_2_Ir(C^N)_2_ and auxiliary ligand triphenylphosphine derivatives shows excellent catalytic ability for C–N bond formation. The catalytic ability of these catalytic systems depends on the structure of the bidentate ligand (C^N) of the metal complex, the type of auxiliary ligand, the ratio of metal complex to auxiliary ligand, and the solvent used. Catalytic system D2/TMPP showed the best performance. Various amines such as aromatic amines, aliphatic amines, primary amines, and secondary amines react well with various alcohols such as aromatic alcohols, aliphatic alcohols, primary alcohols, secondary alcohols, and diols. In 24 C–N bond formation reactions, the D2/TMPP catalytic system showed excellent performance, with an average conversion rate of approximately 97%. N-Heterocycles, such as pyrrolidine derivatives, piperazine derivatives, and large heterocycles (higher than six-membered rings) can be constructed in high yields. These catalytic systems for synthetic strategies could inspire the development of new C–N bond forming reactions as well as pharmaceutical applications for sustainable transformations.

## Experimental

### Materials and methods

Iridium chloride (IrCl_3_, anhydrous) was obtained from the Seedchem Co. All other chemicals including were purchased from Acros and used as received. NMR spectra were measured on a Bruker Advance-400 MHz or a Mercury 300 MHz NMR spectrometer. Elemental analyses (CHN) was obtained from an Elementar vario EL III analyzer. Mass spectrometry was performed on a Finnigan/Thermo Quest MAT 95XL instrument using electron impact ionization for organic compounds and fast atom bombardment for metal complexes.

### Synthesis of benzoxazole derivative ligands (L2–L5)

Benzoxazole derivative ligands L2–L5 were prepared by Philips condensation as follows: In a flask, mix one equivalent of the benzoic acid derivatives (benzoic acid for L2 and L5, 4-chlorobenzoic acid for L3, and 3,5-difluorobenzoic acid for L4) with 1.05 equivalents of the appropriate aminophenol derivatives (aminophenol for L2, L3, and L4, and 2-amino-4-chlorophenol for L5), then polyphosphoric acid (at the ratio of 10 g polyphosphoric acid/mmol of benzoic acid derivatives) were added to this mixture. The mixture was reacted at 140 °C for 24 hours. After cooling to room temperature, the mixture was slowly poured into pure water and stirred thoroughly; the precipitate was collected by filtration, washed with pure water, and dried to obtain a crude product. The crude product was purified by silica gel column chromatography using hexane–dichloromethane as the eluent to obtain the corresponding ligand. Structural data for benzoxazole derivative ligands is shown in the ESI.†

### Synthesis of dinuclear iridium precursors (C^N)2Ir(μ-Cl)2Ir(C^N)2 (D1–D5)

Synthesis of cyclometalated Ir(iii) chloride-bridged dimers (D1–D5) based on previous paper.^40,41^ In a flask, mix the benzoxazole derivatives (L2–L5) with iridium trichloride and a mixed solvent of 2-ethoxyethanol and water (3 : 1, v/v). The ratio of reactants is 1.0 mmol iridium trichloride/2.5 mmol benzoxazole derivative/20 ml mixed solvent. The mixture was reacted at 110 °C for 24 hours under nitrogen. After cooling to room temperature, the mixture was poured into pure water, the dimer precipitate was filtered out, washed with deionized water and ethanol, and then dried in a vacuum oven at 50 °C. Structural data for dinuclear iridium precursors is shown in the ESI.†

### General procedure for *N*-alkylation reaction

In a Schlenk tube, mix the amine with the alcohol, dinuclear iridium precursor, and auxiliary ligand. The ratio of reactants is 1.0 mmol amine/2.0 mmol alcohol/0.015 mmol dinuclear iridium precursor/0.03 mmol auxiliary ligand. The mixture was reacted at 160 °C for 24 hours under nitrogen. The crude product is purified by column chromatography, using dichloromethane/*n*-hexane or methanol/dichloromethane as the eluent to obtain the pure product. The desired *N*-alkylation products were thoroughly characterized by ^1^H, ^13^C NMR and MS spectra. Structural data for *N*-alkylation products are shown in the ESI.†

### X-Ray crystal structure data, NMR and HMS data for Cat. 1–Cat. 7

Catalyst 1–7 was isolated from the catalytic system and crystallized by solvent dispersion using dichloromethane/*n*-hexane. The diffraction data of Cat. 1–Cat. 7 were collected on a Bruker SMART APEX CCD diffractometer with graphite-monochromatized Mo Kα X-ray radiation (*λ* = 0.71073 Å) at room temperature. All the calculations for the structure determination were carried out using SHELXTL package (version 5.1). Initial atomic positions were located by Patterson methods using XS, and the structures of the complexes were refined by the least-square method using SHELXL-97.^42,43^ Basic information pertaining to crystal parameters and structure refinement are summarized in Tables S1 and S3–S4.† Selected bond distances and angles are listed in Tables S2 and S5.† Structural data including ^1^H, ^13^C NMR and MS spectra for Cat. 1–Cat. 7 are shown in the ESI.†

### Density functional theory calculation

The Gaussian 03 software package was used to perform theoretical calculations using Becke's C, H, F, O and N three-parameter B3LYP and the LANL2DZ basis set of the Ir element.^44^ Perform a vibration analysis to classify stationary points as local minima. All energies given are zero-point corrected. Partial charges are calculated in terms of Mulliken, APT (Atomic Polar Tensor) and NPA (Natural Group Number). The atomic coordinates of Cat. 2 and Cat. 4–Cat. 7 used for the calculations are shown in ESI, Tables S7–S12.† The theoretical calculation data of Cat. 2 and Cat. 4–Cat. 7, including the orbital distribution and composition of HOMO and LUMO, are shown in Fig. S1.†

## Author contributions

Yi-Sheng Chen and Siang-Yu Chiu: investigation. S Chia-Ying Li: data curtion, validation. Tsun-Ren Chen and Jhy-Der Chen: writing – review & editing, supervision.

## Conflicts of interest

There are no conflicts to declare.

## Supplementary Material

RA-013-D3RA07184G-s001

RA-013-D3RA07184G-s002

## References

[cit1] He S., Xiao J., Dulcey A. E., Lin B., Rolt A., Hu Z., Hu X., Wang A. Q., Xu X., Southall N., Ferrer M., Zheng W., Liang T. J., Marugan J. J. (2016). Discovery, Optimization, and Characterization of Novel Chlorcyclizine Derivatives for the Treatment of Hepatitis C Virus Infection. J. Med. Chem..

[cit2] Das A., Choi A., Coldham I. (2023). Photocatalysis and Kinetic Resolution by Lithiation to Give Enantioenriched 2-Arylpiperazines. Org. Lett..

[cit3] Tahirovic Y. A., Truax V. M., Wilson R. J., Jecs E., Nguyen H. H., Miller E. J., Kim M. B., Kuo K. M., Wang T., Sum C. S., Cvijic M. E., Schroeder G. M., Wilson L. J., Liotta D. C. (2018). Discovery of *N*-Alkyl Piperazine Side Chain Based CXCR4 Antagonists with Improved Drug-like Properties. ACS Med. Chem. Lett..

[cit4] Leng L., Ready J. M. (2020). Photocatalytic α-Alkylation of Amines with Alkyl Halides. ACS Catal..

[cit5] Peacock D. M., Roos C. B., Hartwig J. F. (2016). Palladium-Catalyzed Cross Coupling of Secondary and Tertiary Alkyl Bromides with a Nitrogen Nucleophile. ACS Cent. Sci..

[cit6] Yang Y.-S., Shen Z.-L., Loh T.-P. (2009). Indium (Zinc)–Copper-Mediated Barbier-Type Alkylation Reaction of Nitrones in Water: Synthesis of Amines and Hydroxylamines. Org. Lett..

[cit7] Powell D. A., Ramsden P. D., Batey R. A. (2003). Phase-Transfer-Catalyzed Alkylation of Guanidines by Alkyl Halides under Biphasic Conditions: A Convenient Protocol for the Synthesis of Highly Functionalized Guanidines. J. Org. Chem..

[cit8] Zhang Y.-F., Dong X.-Y., Cheng J.-T., Yang N.-Y., Wang L.-L., Wang F.-L., Luan C., Liu J., Li Z.-L., Gu Q.-S., Liu X.-Y. (2021). Enantioconvergent Cu-Catalyzed Radical C–N Coupling of Racemic Secondary Alkyl Halides to Access α-Chiral Primary Amines. J. Am. Chem. Soc..

[cit9] Mitsudo K., Shimohara S., Mizoguchi J., Mai H., Suga S. (2012). Synthesis of Nitrogen-Bridged Terthiophenes by Tandem Buchwald–Hartwig Coupling and Their Properties. Org. Lett..

[cit10] Navarro M., Alférez M. G., de Sousa M., Mira-Pizarro J., Campos J. (2022). Dicoordinate Au(i)–Ethylene Complexes as Hydroamination Catalysts. ACS Catal..

[cit11] Zhang L., Wang A., Miller J. T., Liu X., Yang X., Wang W., Li L., Huang Y., Mou C.-Y., Zhang T. (2014). Efficient and Durable Au Alloyed Pd Single-Atom Catalyst for the Ullmann Reaction of Aryl Chlorides in Water. ACS Catal..

[cit12] Wong C. M., McBurney R. T., Binding S. C., Peterson M. B., Gonçales V. R., Gooding J. J., Messerle B. A. (2017). Iridium(iii) homo- and heterogeneous catalysed hydrogen borrowing C–N bond formation. Green Chem..

[cit13] Yan T., Feringa B. L., Barta K. (2016). Benzylamines *via* Iron-Catalyzed Direct Amination of Benzyl Alcohols. ACS Catal..

[cit14] Wang B., Li M., Zhang S., Wu H., Liao Y., Hu L. (2023). Synergistic effect between Co single atoms and nanoparticles enables selective synthesis of bio-based benzimidazoles. Appl. Catal., B.

[cit15] Meng Y., Jian Y., Li J., Wu H., Zhang H., Saravanamurugan S., Yang S., Hu L. (2023). Surface-active site engineering: Synergy of photo- and supermolecular catalysis in hydrogen transfer enables biomass upgrading and H_2_ evolution. Chem. Eng. J..

[cit16] Zou Q., Wang C., Smith J., Xue D., Xiao J. (2015). Alkylation of Amines with Alcohols and Amines by a Single Catalyst under Mild Conditions. Chem.–Eur. J..

[cit17] Kawahara R., Fujita K., Yamaguchi R. (2010). Multialkylation of Aqueous Ammonia with Alcohols Catalyzed by Water-Soluble Cp*Ir–Ammine Complexes. J. Am. Chem. Soc..

[cit18] Shan S. P., Xiaoke X., Gnanaprakasam B., Dang T. T., Ramalingam B., Huynh H. V., Seayad A. M. (2015). Benzimidazolin-2-ylidene N-heterocyclic carbene complexes of ruthenium as a simple catalyst for the *N*-alkylation of amines using alcohols and diols. RSC Adv..

[cit19] Jumde V. R., Gonsalvi L., Guerriero A., Peruzzini M., Taddei M. (2015). A Ruthenium-Based Catalytic System for a Mild Borrowing-Hydrogen Process. Eur. J. Org Chem..

[cit20] Rösler S., Ertl M., Irrgang T., Kempe R. (2015). Cobalt-Catalyzed Alkylation of Aromatic Amines by Alcohols. Angew. Chem., Int. Ed..

[cit21] Quintard A., Rodriguez J. (2016). A Step into an eco-Compatible Future: Iron- and Cobalt-catalyzed Borrowing Hydrogen Transformation. ChemSusChem.

[cit22] Shimizu N. K.-I., Kon K., Onodera W., Yamazaki H., Kondo J. N. (2013). Heterogeneous Ni Catalysts for *N*-Alkylation of Amines with Alcohols. ACS Catal..

[cit23] Satyanarayana P., Reddy G. M., Maheswaran H., Kantam M. L. (2013). Tris(acetylacetonato)rhodium(iii)-Catalyzed α-Alkylation of Ketones, β-Alkylation of Secondary Alcohols and Alkylation of Amines with Primary Alcohols. Adv. Synth. Catal..

[cit24] Zhang Y. Q. X., Qi X., Cui X., Shi F., Deng Y. (2011). Palladium catalyzed *N*-alkylation of amines with alcohols. Tetrahedron Lett..

[cit25] Dang T. T., Shan S. P., Ramalingam B., Seayad A. M. (2015). An efficient heterogenized palladium catalyst for *N*-alkylation of amines and α-alkylation of ketones using alcohols. RSC Adv..

[cit26] Abdukader A., Jin H., Cheng Y., Zhu C. (2014). Rhenium-Catalyzed Amination of Alcohols by Hydrogen Transfer Process. Tetrahedron Lett..

[cit27] Shimizu K.-I., Imaiida N., Kon K., Siddiki H., Satsuma A. (2013). Heterogeneous Ni Catalysts for *N*-Alkylation of Amines with Alcohols. ACS Catal..

[cit28] Pretorius R., Olguín J., Albrecht M. (2017). Carbohydrate-Functionalized 1,2,3-Triazolylidene Complexes for Application in Base-Free Alcohol and Amine Oxidation. Inorg. Chem..

[cit29] Zhao G., Hu D., Zhou S., Zhang J., Wang L. (2019). Supported CuNi Alloy Catalyzed *N*-Alkylation of Bioderived 2,5-Dihydroxymethylfuran With Aniline. Ind. Eng. Chem. Res..

[cit30] Şahin Z., Gürbüz N., Özdemir I., Şahin O., Büyükgüngör O., Achard M., Bruneau C. (2015). *N*-Alkylation and *N*,*C*-Dialkylation of Amines with Alcohols in the Presence of Ruthenium Catalysts with Chelating N-Heterocyclic Carbene Ligands. Organometallics.

[cit31] Celaje J. J. A., Zhang X., Zhang F., Kam L., Herron J. R., Williams T. J. (2017). A Base and Solvent-Free Ruthenium-Catalyzed Alkylation of Amines. ACS Catal..

[cit32] Liu J., Chan A. K.-W., Ng M., Hong E. Y.-H., Wu N. M.-W., Wu L., Yam V. W.-W. (2019). Synthesis, Characterization, and Photochromic Studies of Cyclometalated Iridium(iii) Complexes Containing a Spironaphthoxazine Moiety. Organometallics.

[cit33] Du B.-S., Lin C.-H., Chi Y., Hung J.-Y., Chung M.-W., Lin T.-Y., Lee G.-H., Wong K.-T., Chou P.-T., Hung W.-Y., Chiu H.-C. (2010). Diphenyl(1-naphthyl)phosphine Ancillary for Assembling of Red and Orange-Emitting Ir(iii) Based Phosphors; Strategic Synthesis, Photophysics, and Organic Light-Emitting Diode Fabrication. Inorg. Chem..

[cit34] Chen T.-R., Chen Y.-S., Li C.-Y., Lin Y.-H., Chen Y.-T. (2022). Spontaneous Release of Metalloradicals and Coordinatively Unsaturated Species in Asymmetric Iridium Dimers to Promote C–N Bond Formation. Inorganics.

[cit35] Chen T.-R., Chen Y.-T., Chen Y.-S., Lee W.-J., Lin Y.-H., Wang H.-C. (2022). Iridium/graphene nanostructured catalyst for the *N*-alkylation of amines to synthesize nitrogen-containing derivatives and heterocyclic compounds in a green process. RSC Adv..

[cit36] Balamurugan G., Ramesh R., Malecki J. G. (2020). Nickel(ii)−N^Λ^N^Λ^O Pincer Type Complex-Catalyzed *N*-alkylation of Amines with Alcohols *via* the Hydrogen Autotransfer Reaction. J. Org. Chem..

[cit37] Bains A. K., Kundu A., Yadav S., Adhikari D. (2019). Borrowing Hydrogen-Mediated *N*-Alkylation Reactions by a Well-Defined Homogeneous Nickel Catalyst. ACS Catal..

[cit38] Hashimoto K., Ide S., Arata M., Nakata A., Ito A., Ito T. K., Kudo N., Lin B., Nunomura K., Tsuganezawa K., Yoshida M., Nagaoka Y., Sumiyoshi T. (2022). Discovery of Benzylpiperazine Derivatives as CNS-Penetrant and Selective Histone Deacetylase 6 Inhibitors. ACS Med. Chem. Lett..

[cit39] Javier S.-C., Pablo M.-A., Margarita V.-H., Ana S.-., Cela J. I., Antonio J. M.-L., Pachon J., Ferno I.-G., Manuel J. V.-P. (2016). New 4-Acyl-1-phenylaminocarbonyl-2-phenylpiperazine Derivatives as Potential Inhibitors of Adenovirus Infection. Synthesis, Biological Evaluation, and Structure–activity Relationships. J. Med. Chem..

[cit40] Chen T.-R. (2008). Synthesis and characterization of cyclometalated iridium(iii) complexes containing benzoxazole derivatives and different ancillary ligands. J. Organomet. Chem..

[cit41] Lee H.-P., Hsu Y.-F., Chen T.-R., Chen J.-D., Chen K. H.-C., Wang J.-. (2009). A Novel Cyclometalated Dimeric Iridium Complex, [(dfpbo)_2_Ir]_2_ [dfpbo = 2-(3,5-Difluorophenyl)benzoxazolato-*N*,*C*^2^], Containing an Unsupported Ir^II^–Ir^II^ Bond. Inorg. Chem..

[cit42] Bruker A.X.S. , APEX2, V2008.6, SADABS V2008/1, SAINT V7.60A, SHELXTL V6.14, Bruker AXS Inc., Madison, WI, USA, 2008

[cit43] Sheldrick G. M. (2008). A short history of SHELX. Acta Crystallogr., Sect. A: Found. Crystallogr..

[cit44] DFT (B3LYP/LANL2DZ level) calculation: Frisch M. J.; Trucks G. W.; Schlegel H. B.; Scuseria G. E.; Robb M. A.; Cheeseman J. R.; Montgomery J. A.; Vreven, Jr T.; Kudin K. N.; Burant J. C.; Millam J. M.; Iyengar S. S.; Tomasi J.; Barone V.; Mennucci B.; Cossi M.; Scalmani G.; Rega N.; Petersson G. A.; Nakatsuji H.; Hada M.; Ehara M.; Toyota K.; Fukuda R.; Hasegawa J.; Ishida M.; Nakajima T.; Honda Y.; Kitao O.; Nakai H.; Klene M.; Li X.; Knox J. E.; Hratchian H. P.; Cross J. B.; Adamo C.; Jaramillo J.; Omperts R.; Stratmann R. E.; Yazyev O.; Austin A. J.; Cammi R.; Pomelli C.; Ochterski J. W.; Ayala P. Y.; Morokuma K.; Voth G. A.; Salvador P.; Dannenberg J. J.; Zakrzewski V. G.; Dapprich S.; Daniels A. D.; Strain M. C.; Farkas O.; Malick D. K.; Rabuck A. D.; Raghavachari K.; Foresman J. B.; Ortiz J. V.; Cui Q.; Baboul A. G.; Clifford S.; Cioslowski J.; Stefanov B. B.; Liu G.; Liashenko A.; Piskorz P.; Komaromi I.; Martin R. L.; Fox D. J.; Keith T.; Al-Laham M. A.; Peng C. Y.; Nanayakkara A.; Challacombe M.; Gill P. M. W.; Johnson B.; Chen W.; Wong M. W.; Gonzalez C. and Pople A. Gaussian 03, Revision B.04, Gaussian, Inc, Pittsburgh PA, 2003

