# Supplementary material for: Ligand effect of cyclometallated iridium(iii) complexes on N-alkylation of amines in hydrogen borrowing reactions
Source: RSC Adv. 2023 Oct 31;13(45):31948–61. doi: 10.1039/d3ra07184g (PMC10617371; doi:10.1039/d3ra07184g)
Supplement: RA-013-D3RA07184G-s001 [file RA-013-D3RA07184G-s001.pdf]

Electronic Supplementary Material (ESI) for RSC Advances.
This journal is © The Royal Society of Chemistry 2023

Supporting Information

Ligand Effect of Cyclometallated Iridium (III) Complexes on N-Alkylation of Amines in Hydrogen Borrowing Reactions

Yi-Sheng Chen[a], Siang-Yu Chiu[a], Chia-Ying Li [a], Tsun-Ren Chen* [a], and Jhy-Der Chen [b]

[a] Department of Applied Chemistry, National Pingtung University, Pingtung, Taiwan,90003

[b] Department of Chemistry, Chung Yuan Christian University, Chung-Li, Taiwan, R.O.C.

Contents **Page**

Crystal data for **Cat. 1~7**	S3
Table S1. Crystal data for **Cat. 1** ~ **Cat. 3**	S3
Table S2. Selected bond distance (Å) and bond angles for **Cat. 1~3**	S4
Table S3. Crystal data for **Cat. 4** and **Cat. 5**	S6
Table S4. Crystal data for **Cat. 6** and **Cat. 7**	S7
Table S5. Selected bond distance (Å) and bond angles for **Cat. 4~7**	S8
Figure S1 Theoretical calculation data of **Cat.2** and **4-7**, including the orbital distribution and composition of HOMO and LUMO	S10
Structural data of Benzoxazole Derivative Ligands	S11
Structural data of Dinuclear Iridium Precursors $(C^\wedge N)_2Ir(\mu\text{-}Cl)_2Ir(C^\wedge N)_2$ (**D1~D5**)	S12
Structural Data of Catalysts $(C^\wedge N)_2Ir(Cl)$ (TPP) (Cat.1~7)	S13
Structural data of N-alkylation Products.	S15
The Atomic Coordinates of **Cat. 1~7**	S23
Table S6. The Atomic Coordinates (angstroms) of **Cat. 1**	S23
Table S7. The Atomic Coordinates (angstroms) of **Cat. 2**	S25
Table S8. The Atomic Coordinates (angstroms) of **Cat. 3**	S27
Table S9. The Atomic Coordinates (angstroms) of **Cat. 4**	S29
Table S10. The Atomic Coordinates (angstroms) of **Cat. 5**	S31
Table S11. The Atomic Coordinates (angstroms) of **Cat. 6**	S33
Table S12. The Atomic Coordinates (angstroms) of **Cat. 7**	S35

I. Crystal data for **Cat. 1~7**

Table S1. Crystal data for **Cat. 1** ~ **Cat. 3**

**Cat.**	**1**	**2**	**3**
empirical formula	C_{42} H_{35} Cl_5 Ir N_2 P	C_{50} H_{45} Cl Ir N_2 O_3 P	C_{42} H_{32} Cl_5 F_3 Ir N_2 P
fw	968.14	980.50	1022.11
temp [K]	110(2)	296(2)	296(2)
radiated used (λ[Å])	0.71073	0.71073	0.71073
cryst size [mm^3]	0.66 x 0.56 x 0.54	0.200 x 0.100 x 0.100	0.200 x 0.100 x 0.100
cryst syst	Triclinic	Triclinic	Triclinic
space group	P -1	P-1	P-1
a [Å]	11.0738(5)	12.911(6)	11.250(13)
b [Å]	13.2326(6)	13.195(6)	13.317(16)
c [Å]	13.7902(5)	14.842(7)	14.035(17)
α [deg]	83.693(3)°	82.321(12)°	84.55(3)°.
β [deg]	70.637(3)°	72.051(10)°	74.18(3)°.
γ [deg]	82.745(4)°	63.435(10)°	83.10(3)°.
V [$Å^3$], Z	1886.19(14), 4	2151.4(18) ,2	2004(4) , 2
$ρ_{calcd}$ [$g.cm^{-3}$]	1.705	1.514	1.694
μ [mm^{-1}]	3.970 mm^{-1}	3.247	3.752
F(000)	956	984	1004
scan rang θ [deg]	2.88 to 29.21°	1.442 to 26.000°.	1.511 to 25.998°.
no. of total refins	16612	42883	40353
no. of unique refins	8694 [R(int) = 0.0221]	8448 [R(int) = 0.1296]	7871 [R(int) = 0.1774]
no. of data/restraints/ params	8694 / 0 / 460	8448 / 1 / 523	7871 / 0 / 487
goodness-of-fit on F^2	0.986	0.940	0.973
R_1, wR_2 [$I>σ2(I)$]	0.0217 and 0.0474	R1 = 0.0451, wR2 = 0.0547	R1 = 0.0609, wR2 = 0.0736
R_1, wR_2 (all data)	0.0257 and 0.0479	R1 = 0.0881, wR2 = 0.0629	R1 = 0.1289, wR2 = 0.0882

Table S2. Selected bond distance (Å) and bond angles for **Cat. 1~3**.

**Cat. 1**			
Ir-C(22)	2.004(2)	Ir-N(1)	2.062(2)
Ir-C(11)	2.044(2)	Ir-P	2.4074(6)
Ir-N(2)	2.053(2)	Ir-Cl(1)	2.4919(6)
C(22)-Ir-C(11)	89.14(9)	N(2)-Ir-P	89.94(6)
C(22)-Ir-N(2)	80.64(9)	N(1)-Ir-P	98.60(6)
C(11)-Ir-N(2)	91.70(9)	C(22)-Ir-Cl(1)	172.90(7)
C(22)-Ir-N(1)	93.60(9)	C(11)-Ir-Cl(1)	86.98(7)
C(11)-Ir-N(1)	80.05(9)	N(2)-Ir-Cl(1)	93.53(6)
N(2)-Ir-N(1)	170.06(8)	N(1)-Ir-Cl(1)	91.58(6)
C(22)-Ir-P	94.17(6)	P-Ir-Cl(1)	89.85(2)
C(11)-Ir-P	176.51(7)		
**Cat. 2**			
Ir-C(22)	2.012(5)	Ir-N(1)	2.066(4)
Ir-C(11)	2.049(5)	Ir-P	2.4388(16)
Ir-N(2)	2.058(4)	Ir-Cl	2.4861(15)
C(22)-Ir-C(11)	89.91(18)	N(2)-Ir-P	91.12(12)
C(22)-Ir-N(2)	80.74(19)	N(1)-Ir-P	97.32(13)
C(11)-Ir-N(2)	91.65(18)	C(22)-Ir-Cl	172.91(15)
C(22)-Ir-N(1)	95.85(18)	C(11)-Ir-Cl	87.62(13)
C(11)-Ir-N(1)	80.00(19)	N(2)-Ir-Cl	92.68(12)
N(2)-Ir-N(1)	171.03(16)	N(1)-Ir-Cl	90.27(11)
C(22)-Ir-P	92.27(14)	P-Ir-Cl	90.49(6)
C(11)-Ir-P	176.71(14)		
**Cat. 3**			
Ir-C(12)	2.002(9)	Ir-N(2)	2.064(6)
Ir-C(11)	2.041(8)	Ir-P	2.419(3)
Ir-N(1)	2.064(6)	Ir-Cl(1)	2.499(3)
C(12)-Ir-C(11)	88.1(3)	N(1)-Ir-P	98.36(18)
C(12)-Ir-N(1)	94.2(3)	N(2)-Ir-P	90.42(18)
C(11)-Ir-N(1)	79.8(3)	C(12)-Ir-Cl(1)	172.1(2)
C(12)-Ir-N(2)	79.8(3)	C(11)-Ir-Cl(1)	87.3(2)

C(11)-Ir-N(2)	91.6(3)	N(1)-Ir-Cl(1)	91.29(17)
N(1)-Ir-N(2)	169.7(2)	N(2)-Ir-Cl(1)	93.9(2)
C(12)-Ir-P	94.1(2)	P-Ir-Cl(1)	90.76(8)
C(11)-Ir-P	177.2(3)		

Table S3. Crystal data for **Cat. 4** and **Cat. 5**

**Cat.**	**4**	**5**
empirical formula	C49 H41 Cl5 Ir N2 O5 P	C47 H35 Cl3 Ir N2 O5 P
fw	1138.26	1037.29
temp [K]	293(2)	296(2)
radiated used (λ[Å])	0.71073	0.71073
cryst size [mm^3]	0.800 x 0.300 x 0.200	0.500 x 0.500 x 0.500
cryst syst	Triclinic	Triclinic
space group	P-1	P-1
a [Å]	12.34(2)	12.499(3)
b [Å]	12.49(2)	14.154(3)
c [Å]	16.46(4)	16.193(3)
α [deg]	86.17(5)°.	85.480(6)°.
β [deg]	69.00(5)°.	87.279(6)°.
γ [deg]	88.19(5)°.	65.482(6)°.
V [$Å^3$], Z	2364(8) , 2	2597.9(9) , 2
ρ_{calcd} [$g.cm^{-3}$]	1.599	1.326
μ [mm^{-1}]	3.189	2.795
F(000)	1132	1028
scan rang θ [deg]	1.327 to 25.998°.	1.585 to 26.000°.
no. of total refins	9296	35372
no. of unique refins	9296 [R(int) = ?]	10226 [R(int) = 0.0793]
no. of data/restraints/ params	9296 / 0 / 563	10226 / 0 / 532
goodness-of-fit on F^2	1.060	1.031
R_1, wR_2 [I>σ2(I)]	R1 = 0.0526, wR2 = 0.1074	R1 = 0.0419, wR2 = 0.0914
R_1, wR_2 (all data)	R1 = 0.0804, wR2 = 0.1246	R1 = 0.0616, wR2 = 0.0998

Table S4. Crystal data for **Cat. 6** and **Cat. 7**

**Cat.**	**6**	**7**
empirical formula	C47.60 H38.80 Cl F4 Ir N2 O7.30 P	C47.25 H37.50 Cl3.50 Ir N2 O6 P
fw	1090.23	1076.54
temp [K]	297(2)	296(2) K
radiated used (λ[Å])	0.71073	0.71073 Å
cryst size [mm^3]	0.42 x 0.39 x 0.23	0.300 x 0.100 x 0.100
cryst syst	Orthorhombic	Triclinic
space group	P b c a	P-1
a [Å]	19.1611(8)	a = 13.2859(8)
b [Å]	20.4125(9)	b = 13.4065(8)
c [Å]	23.7193(10)	c = 15.6834(10)
α [deg]	90°.	= 103.2380(15)°
β [deg]	90°.	= 90.1227(16)°
γ [deg]	90°.	= 117.8150(14)°
V [Å^3], Z	9277.2(7) , 8	2385.7(3), 2
ρ_{calcd} [g.cm^{-3}]	1.561	1.499
μ [㎜$^{-1}$]	3.039	3.075
F(000)	4334	1069
scan rang θ [deg]	1.69 to 26.01°.	1.747 to 26.000°.
no. of total refins	50639	33139
no. of unique refins	9119 [R(int) = 0.0427]	9373 [R(int) = 0.1131]
no. of data/restraints/ params	9119 / 0 / 640	9373 / 3 / 550
goodness-of-fit on F^2	1.047	1.005
R_1, wR_2 [I>σ2(I)]	R1 = 0.0324, wR2 = 0.1027	R1 = 0.0602, wR2 = 0.1364
R_1, wR_2 (all data)	R1 = 0.0535, wR2 = 0.1171	R1 = 0.1096, wR2 = 0.1565

Table S5. Selected bond distance (Å) and bond angles for **Cat. 4~7**.

	**Cat. 4**		
Ir-C(1)	2.050(8)	Ir-N(1)	2.095(6)
Ir-C(14)	2.084(9)	Ir-P(1)	2.427(4)
Ir-N(2)	2.091(7)	Ir-Cl(1)	2.463(4)
C(1)-Ir-C(14)	89.6(3)	N(2)-Ir-P(1)	100.2(2)
C(1)-Ir-N(2)	92.8(3)	N(1)-Ir-P(1)	90.9(2)
C(14)-Ir-N(2)	80.2(3)	C(1)-Ir-Cl(1)	174.6(2)
C(1)-Ir-N(1)	81.3(3)	C(14)-Ir-Cl(1)	87.3(2)
C(14)-Ir-N(1)	89.1(3)	N(2)-Ir-Cl(1)	91.10(19)
N(2)-Ir-N(1)	167.8(2)	N(1)-Ir-Cl(1)	94.14(19)
C(1)-Ir-P(1)	94.8(2)	P(1)-Ir-Cl(1)	88.17(11)
C(14)-Ir-P(1)	175.5(2)		

	**Cat. 5**		
Ir-C(14)	2.018(5)	Ir-N(1)	2.226(4)
Ir-N(2)	2.080(4)	Ir-Cl(1)	2.3751(13)
Ir-C(1)	2.106(5)	Ir-P	2.3972(14)
C(14)-Ir-N(2)	80.17(17)	C(1)-Ir-Cl(1)	84.34(12)
C(14)-Ir-C(1)	89.94(18)	N(1)-Ir-Cl(1)	87.71(11)
N(2)-Ir-C(1)	92.07(15)	C(14)-Ir-P	89.93(14)
C(14)-Ir-N(1)	167.63(17)	N(2)-Ir-P	90.24(10)
N(2)-Ir-N(1)	95.63(14)	C(1)-Ir-P	177.63(12)
C(1)-Ir-N(1)	78.53(16)	N(1)-Ir-P	101.77(11)
C(14)-Ir-Cl(1)	95.67(15)	Cl(1)-Ir-P	93.31(5)
N(2)-Ir-Cl(1)	174.53(10)		

	**Cat. 6**		
Ir-C(26)	2.047(4)	Ir-N(1)	2.081(4)
Ir-N(2)	2.049(4)	Ir-P	2.4145(14)
Ir-C(13)	2.080(5)	Ir-Cl	2.4465(12)
C(26)-Ir-N(2)	79.42(17)	C(13)-Ir-P	173.97(14)
C(26)-Ir-C(13)	89.23(19)	N(1)-Ir-P	99.40(11)
N(2)-Ir-C(13)	92.4(2)	C(26)-Ir-Cl	170.24(13)

C(26)-Ir-N(1)	96.53(17)	N(2)-Ir-Cl	94.08(12)
N(2)-Ir-N(1)	171.52(16)	C(13)-Ir-Cl	83.73(14)
C(13)-Ir-N(1)	80.07(19)	N(1)-Ir-Cl	88.93(11)
C(26)-Ir-P	96.80(13)	P-Ir-Cl	90.25(4)
N(2)-Ir-P	88.52(12)		

Cat. 7

Ir-C(13)	2.033(9)	Ir-N(2)	2.067(7)
Ir-N(1)	2.046(7)	Ir-P	2.416(2)
Ir-C(26)	2.062(10)	Ir-Cl(1)	2.480(2)
C(13)-Ir-N(1)	80.7(3)	C(26)-Ir-P	174.3(3)
C(13)-Ir-C(26)	88.3(4)	N(2)-Ir-P	100.5(2)
N(1)-Ir-C(26)	90.5(3)	C(13)-Ir-Cl(1)	171.6(3)
C(13)-Ir-N(2)	91.3(3)	N(1)-Ir-Cl(1)	93.4(2)
N(1)-Ir-N(2)	167.5(3)	C(26)-Ir-Cl(1)	85.8(3)
C(26)-Ir-N(2)	79.5(3)	N(2)-Ir-Cl(1)	93.40(19)
C(13)-Ir-P	97.3(3)	P-Ir-Cl(1)	88.58(8)
N(1)-Ir-P	90.2(2)		

Cat.	**2**	**4**	**5**	**6**	**7**
LUMO	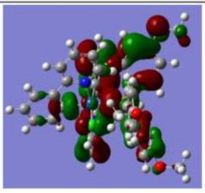<br>Composition<br>5.19% Ir<br>35.65% Ph<br>17.04% Py	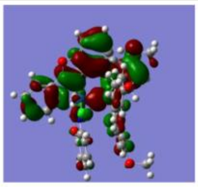<br>Composition<br>5.50% Ir<br>22.64% Ph<br>17.47% Ox	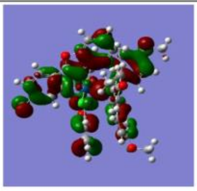<br>Composition<br>5.48% Ir<br>29.09% Ph<br>18.94% Ox	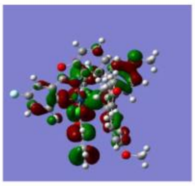<br>Composition<br>5.30% Ir<br>32.06% Ph<br>18.21% Ox	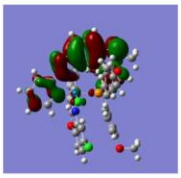<br>Composition<br>3.02% Ir<br>19.47% Ph<br>20.50% Ox
HOMO	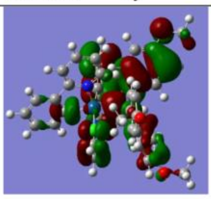<br>Composition<br>5.19% Ir<br>34.92% Ph<br>16.86% C^N	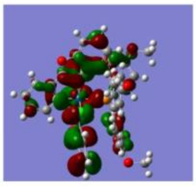<br>Composition<br>4.75% Ir<br>29.86% Ph<br>21.72% Ox	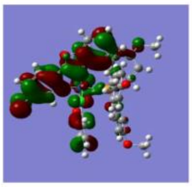<br>Composition<br>4.19% Ir<br>32.35% Ph<br>24.05% Ox	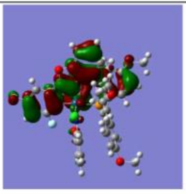<br>Composition<br>3.97% Ir<br>33.02% Ph<br>17.71% Ox	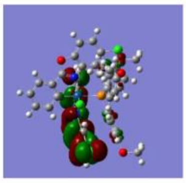<br>Composition<br>3.33% Ir<br>22.72% Ph<br>27.43% Ox

Ph: The phenyl group of the C^N ligand
Py: The pyridyl of C^N ligands
Ox : The benzoxazole groups of C^N ligands

Figure S1. Theoretical calculation data of **Cat.2** and **4**-**7**, including the orbital distribution and composition of HOMO and LUMO

II. Structural Data of Benzoxazole Derivative Ligands

2-phenylbenzoxazole (pbo) (**L2**): 80% yield. 1H NMR (300 MHz, CDCl3, 298 K; δ (ppm)): 8.28−8.25 (m, 2H), 7.80−7.76 (m, 1H), 7.60−7.56 (m, 1H), 7.53−7.51 (m, 3H), 7.37−7.33 (m, 2H). 13C NMR (75 MHz, CDCl3, 298 K; δ (ppm)): δ163.2, 151.0, 142.3, 131.7, 129.1, 127.8, 127.4, 125.3, 124.8, 120.263, 110.8. Anal. Calcd for $C_{13}H_9NO$ (MW = 195.22): C, 79.98; H, 4.65; N, 7.18. Found: C, 79.99; H, 4.65; N, 7.20%. MS (FAB; m/z): 195.0686

2-(4-Chlorophenyl)benzoxazole (cpbo) (**L3**): 75% yield. 1H NMR (300 MHz, CDCl3, 298 K; δ (ppm)): 8.17 (d, J = 8.7 Hz, 2H), 7.77−7.74 (m, 1H), 7.57−7.54 (m, 1H), 7.48 (d, J = 8.7 Hz, 2H), 7.38−7.33 (m, 2H). 13C NMR (75 MHz, CDCl3, 298 K; δ (ppm)): 162.2, 150.9, 142.2, 137.9, 129.4, 129.0, 125.8, 125.5, 124.9, 120.3, 110.8. Anal. Calcd for $C_{13}H_8NOCl$ (MW = 229.67): C, 67.98; H, 3.51; N, 6.10. Found: C, 67.98; H, 3.53; N, 6.11%. MS (FAB; m/z):229.0303

2-(3.5-Difluorophenyl)benzoxazole (fpbo) (**L4**): 88% yield. 1H NMR (300 MHz, CDCl3, 298 K; δ (ppm)): 7.80−7.74 (m, 3H), 7.59−7.56 (m, 1H), 7.41−7.36 (m, 2H), 7.00−6.96 (m, 1H). 13C NMR (75 MHz, CDCl3, 298 K; δ (ppm)): 165.1, 164.9, 161.8, 161.7, 160.9, 151.0, 141.9, 130.4, 130.3, 130.1, 126.1, 125.2, 120.6, 110.9, 110.8, 110.7, 110.6, 107.3, 107.0, 106.7. Anal. Calcd for $C_{13}H_7NO\ F_2$ (MW = 231.20): C, 67.53; H, 3.05; N, 6.06. Found: C, 67.50; H, 3.08; N, 5.99%. MS (FAB; m/z): 231.0496

2-Phenyl-5-chlorobenzoxazole (pcbo) (**L5**): 75% yield. 1H NMR (300 MHz, CDCl3, 298 K; δ (ppm)): 8.23−8.20 (m, 2H), 7.66 (d, J = 8.7 Hz, 1H), 7.58 (d, J = 1.8 Hz, 1H), 7.55−7.49 (m, 3H), 7.32 (dd, J = 8.7, 1.8 Hz, 1H). 13C NMR (75 MHz,

CDCl3, 298 K; δ (ppm)): 163.9, 151.1, 141.1, 132.0, 130.9, 129.2, 127.8, 126.9, 125.5, 120.7, 111.4. Anal. Calcd for $C_{13}H_8NOCl$ (MW = 229.67): C, 67.98; H, 3.51; N, 6.10. Found: C, 67.91; H, 3.52; N, 6.09%. MS (FAB; m/z): 229.0300

III. Structural Data of Dinuclear Iridium Precursors $(C^\wedge N)_2Ir(\mu\text{-}Cl)_2Ir(C^\wedge N)_2$ (D1~D5)

$(pp)_2Ir(\mu\text{-}Cl)_2Ir(pp)_2$ (**D**1): 85% yield. 1H NMR (300 MHz, DMSO-d6, 298 K; δ (ppm)): 8.39−8.35 (m, 4H), 8.18−8.14 (m, 4H), 7.53−7.47 (m, 4H) , 7.44−7.35 (m, 6H) , 7.16−8.12 (m, 2H) , 6.93−6.87 (m, 2H). Anal. Calcd for $C_{44}H_{22}N_4Cl_2Ir_2$ (MW = 1072.09): C, 49.29; H, 3.01; N, 5.23%. Found: C, 49.67; H, 3.08; N, 5.31%. MS (FAB; m/z): 1070.9610

$(pbo)_2Ir(\mu\text{-}Cl)_2Ir(pbo)_2$ (**D2**): 90% yield. 1H NMR (300 MHz, DMSO-d6, 298 K; δ (ppm)): 7.78−7.76 (m, 4H), 7.75−7.73 (m, 10H), 7.52−7.49 (m, 4H), 7.43−7.41 (m, 4H), 7.40−7.36 (m, 10H). Anal. Calcd for $C_{52}H_{32}N_4O_4Cl_2Ir_2$ (MW = 1232.17): C, 50.69; H, 2.61; N, 4.55%. Found: C, 50.67; H, 2.63; N, 4.51%. MS (FAB; m/z): 1231.8585

$(cpbo)_2Ir(\mu\text{-}Cl)_2Ir(cpbo)_2$ (**D3**): 88% yield. 1H NMR (300 MHz, DMSO-d6, 298 K; δ (ppm)): 8.76−8.73 (m, 2H), 8.09−8.54 (m, 4H), 7.95 (d, J = 8.4 Hz, 2H), 7.84−7.60 (m, 8H), 7.48 (dd, J = 8.2, 2.1 Hz, 2H), 7.38 (t, J = 8.4 Hz, 2H), 7.14 (t, J = 8.4 Hz, 2H), 7.03 (dd, J = 8.1, 1.8 Hz, 2H), 6.40 (d, J = 7.8 Hz, 2H), 6.0 (d, J = 2.1 Hz, 2H). Anal. Calcd for $C_{52}H_{28}N_4O_4Cl_6Ir_2$ (MW = 1369.98): C, 45.59; H, 2.06; N, 4.09%. Found: C, 45.67; H, 2.10; N, 4.11%. MS (FAB; m/z): 1367.9515

$(fpbo)_2Ir(\mu\text{-}Cl)_2Ir(fpbo)_2$ (**D4**): 78% yield. 1H NMR (300 MHz, DMSO-d6, 298 K; δ (ppm)): 8.18 (d, J = 8.18, 8.1 Hz, 4H), 7.31 (dd, J = 7.6, 2.7 Hz, 4H), 7.22−7.12 (m, 8H),

6.99 (dt, J = 7.1, 1.5 Hz, 4H), 6.14 (dt, J = 9.6, 2.7 Hz, 4H). 13C NMR (75 MHz, CDCl3, 298 K; δ (ppm)): 174.9, 169.2, 169.1, 167.5, 167.4, 160.3, 160.2, 158.7, 158.6, 149.1, 140.8, 133.6, 133.5, 133.4, 133.3, 125.1, 124.4, 118.0, 115.3, 115.0, 110.9, 108.6, 108.4, 107.2, 107.0, 106.8, 31.8, 31.1, 22.8, 14.3. Anal. Calcd for $C_{52}H_{24}N_4O_4Cl_2F_8Ir_2$ (MW = 1376.12): C, 45.38; H, 1.76; N, 4.07%. Found: C, 45.80; H, 1.80; N, 4.03%. MS (FAB; m/z): 1376.0319.

$(pcbo)_2Ir(\mu\text{-}Cl)_2Ir(pcbo)_2$ (D5): 80% yield. 1H NMR (300 MHz, DMSO-d6, 298 K; δ (ppm)): 8.22 (d, J = 1.8 Hz, 4H), 7.65 (dd, J = 7.8, 1.2 Hz, 4H), 7.36 (d, J = 9 Hz, 4H), 7.1 (dd, J = 8.7, 2.1 Hz, 4H), 6.87 (dt, J = 7.4, 1.2 Hz, 4H), 6.68 (dt, J = 7.4, 1.2 Hz, 4H), 6.17 (d, J = 7.8 Hz, 4H). Anal. $C_{52}H_{28}N_4O_4Cl_6Ir_2$ (MW = 1369.98): C, 45.59; H, 2.06; N, 4.09%. Found: C, 45.38; H, 2.08; N, 4.05%. MS (FAB; m/z): 1367.9504.

IV. Structural Data of Catalysts $(C^\wedge N)_2Ir(Cl)$ (TPP) (Cat.1~7)

$(pp)_2Ir(Cl)(TPP)$ (Cat. 1): 1H NMR (300 MHz, DMSO-d6, 298 K; δ (ppm)): 9.194(d, J=6Hz, 1H), 8.849(d, J=5.4Hz, 1H), 7.883(d, J=7.8Hz, 1H), 7.565(d, J=7.8Hz, 1H), 7.083(t, J=6.9Hz, 4H), 6.832(t, J=7.2Hz, 1H), 6.803(t, J=5.7Hz, 1H), 6.693(t, J=5.7Hz, 1H), 5.888(d, J=7.8Hz, 1H), 5.835(d, J=6.75Hz, 1H). Anal. Calcd for $C_{40}H_{31}N_2ClPIr$ (MW = 798.3282): C, 60.18; H, 3.91; N, 3.51%. Found: C, 60.67; H, 3.85; N, 3.49%. HMS (FAB; m/z): 798.2241

$(pp)_2Ir(Cl)(TMPP)$ (Cat. 2): 1H NMR (300 MHz, DMSO-d6, 298 K; δ (ppm)): 9.306(d, J=5.4Hz, 1H), 8.523(d, J=5.4Hz, 1H), 7.878(d, J=8.4Hz, 1H), 7.563(d, J=8.4Hz, 1H), 7.535(d, J=7.8Hz, 1H), 7.473(t, J=7.8Hz,, 1H), 7.428(d, J=7.2Hz, 1H), 7.129(t, J=9Hz, 3H), 6.836~6.792(m, 1H), 6.611(d, J=7.2Hz, 3H), 5.929(d, J=7.8Hz, 1H) Anal. Calcd

for $C_{43}H_{37}N_2O_3ClPIr$ (MW = 888.4043): C, 58.13; H, 4.20; N, 3.15%. Found: C, 58.67; H, 4.30; N, 3.17%. HMS (FAB; m/z): 888.2559

(pp)$_2$Ir(Cl)(TFPP) (**Cat. 3**): 1H NMR (300 MHz, DMSO-d6, 298 K; δ (ppm)): 8.715(d, J=5.4Hz, 1H), 7.907(d, J=8.4Hz, 1H), 7.725(t, J=7.8Hz, 1H), 7.576(d, J=9.6Hz, 1H), 7.529(t, J=7.8Hz, 1H), 7.496(d, J=4.2Hz, 1H), 7.240~7.180(m, 4H), 6.576(t, J=8.1Hz, 1H), 5.819(t, J=6.6Hz, 1H) Anal. Calcd for $C_{40}H_{28}N_2ClF_3PIr$ (MW = 852.2999): C, 56.37; H, 3.31; N, 3.29%. Found: C, 56.67; H, 3.30; N, 3.31%. HMS (FAB; m/z): 852.1959.

(pbo)$_2$Ir(Cl)(TMPP) (**Cat. 4**): 1H NMR (300 MHz, DMSO-d6, 298 K; δ (ppm)): 8.585(d, J=8.4Hz, 1H), 7.739(d, J=7.8Hz, 1H), 7.637(d, J=8.4Hz, 1H), 7.559(d, J=7.8Hz, 1H), 7.349(d, J=8.4Hz, 1H), 7.301(t, J=8.1Hz, 1H), 7.111(t, J=7.8Hz, 1H), 7.067(d, J=7.8Hz, 1H), 6.815(t, J=7.5Hz, 1H), 6.699(t, J=6.4Hz, 1H), 6.479(d, J=7.2Hz, 3H). Anal. Calcd for $C_{47}H_{37}N_2O_5ClPIr$ (MW = 968.4451): C, 58.29; H, 3.85; N, 2.89%. Found: C, 58.67; H, 3.80; N, 2.81%. HMS (FAB; m/z): 968.2458

(cpbo)$_2$Ir(Cl)(TMPP) (**Cat. 5**): 1H NMR (300 MHz, DMSO-d6, 298 K; δ (ppm)): 7.711(d, J=8.4Hz, 1H), 7.688(d, J=1.8Hz, 1H), 7.674(d, J=1.8Hz, 1H), 7.456(d, J=7.8Hz, 1H), 7.104(t, J=7.8Hz, 1H), 7.067(d, J=4.2Hz, 1H), 6.968(t, J=8.1Hz, 1H), 6.942(t, J=8.1Hz, 1H), 6.883(d, J=4.2Hz, 1H), 6.507(d, J=8.1Hz, 3H), 6.286(d, J=3.9Hz, 1H), 6.042(d, J=7.8Hz, 1H). Anal. Calcd for $C_{47}H_{35}N_2O_5Cl_3PIr$ (MW = 1037.3355): C, 54.42; H, 3.40; N, 2.70%. Found: C, 54.67; H, 3.30; N, 2.75%. HMS (FAB; m/z): 1037.1667

(fpbo)$_2$Ir(Cl)(TMPP) (**Cat. 6**): 1H NMR (300 MHz, DMSO-d6, 298 K; δ (ppm)): 8.529(d,

J=7.8Hz, 1H), 7.626(d, J=8.4Hz, 1H), 7.428(d, J=3.6Hz, 1H), 7.305(t, J=7.5Hz, 2H), 7.150(d, J=5.1Hz, 1H), 7.063(t, J=7.5Hz, 2H), 6.512(s, 3H), 6.297~6.260(m, 2H) Anal. Calcd for $C_{47}H_{33}N_2O_5ClF_4PIr$ (MW = 1040.4074): C, 54.26; H, 3.20; N, 2.69%. Found: C, 54.37; H, 3.18; N, 2.71%. HMS (FAB; m/z): 1040.2081

(pcbo)$_2$Ir(Cl)(TMPP) (**Cat. 7**): 1H NMR (300 MHz, DMSO-d6, 298 K; δ (ppm)): 8.490 (d, J=1.8Hz, 1H), 7.745(d, J=7.8Hz, 1H), 7.620(d, J=3.9Hz, 1H), 7.577(d, J=9Hz, 1H), 7.348(s, 1H), 7.302~7.240(m, 3H), 6.968(t, J=7.5Hz, 2H), 6.764~6.739(m, 5H) Anal. Calcd for $C_{47}H_{35}N_2O_5Cl_3PIr$ (MW = 1037.3355): C, 54.42; H, 3.40; N, 2.70%. Found: C, 54.47; H, 3.50; N, 2.65%. MS (FAB; m/z): 1037.2559

V. Structural Data of N-alkylation Products.

***N*-benzylaniline (P$_1$)**:^{1}H NMR (d-chloroform, 600MHz) = 7.38-7.33 (m, Ar, 4H), 7.28-7.25 (m, Ar, 1H), 7.19-7.16 (m, Ar, 2H), 6.73-6.68 (t, Ar, 1H), 6.65-6.63 (d, Ar, 2H, JHH = 7.8Hz), 4.33 (s, CH2, 2H), 4.02 (br. s, NH, 1H). ^{13}C NMR (d-Chloroform, 150MHz) = 148.2, 139.4, 129.3, 128.7, 127.5, 127.3, 117.6, 112.8, 48.3. MS (ESI/CI): m/z =183.02, Calculated for C13H13N: 183.26 g/mol.

***N*-(*p*-chlorobenzyl)aniline (P$_2$)**: ^{1}H NMR (d-chloroform, 600MHz) = 7.29 (s, 3H, Ar), 7.17-7.11 (m, 2H, Ar), 6.73-6.68 (m, 1H, Ar), 6.58-6.59 (m, 2H, Ar), 4.28 (d, 2H, JHH = 5.4Hz), 4.05 (br. s, NH, 1H). ^{13}C NMR (d-Chloroform, 150MHz) = 147.8, 137.8, 132.9, 129.3,128.8, 128.7, 117.8, 112.9, 47.506. MS (ESI/CI): m/z = 216.98, Calculated for

$C_{13}H_{12}NCl^+$ $[M]^+$: 217.68 g/mol.

***N*-(4-methoxybenzyl)aniline (P₃):** ^{1}H NMR (d-chloroform, 600MHz) = 7.29-7.27 (d, JHH = 8.4Hz, 2H, Ar), 7.18-7.15 (m, 2H, Ar), 6.88-6.86 (m, 2H, Ar), 6.72-6.69 (t, 1H, Ar), 6.63-6.62 (m, 2H, Ar), 4.24 (br. s, NH, 1H), 3.91 (d, JHH = 13.2Hz, 1H) , 3.79 (s, 3H). .^{13}C NMR (d-Chloroform, 150MHz) = 158.9, 148.2, 131.4, 129.3, 128.8, 117.5, 113.8, 112.8, 55.3, 47.8. MS (ESI/CI): m/z = 213.21, Calculated for $C_{14}H_{15}NO^+$ $[M]^+$: 213.28 g/mol.

***N*-benzyl, *N*-(4-chlorophenyl)amine (P4) :** ^{1}H NMR (d-chloroform, 600MHz) = 7.34-7.20 (m, Ar, 4H), 7.11-7.07 (m, Ar, 2H), 6.55-6.5102 (m, Ar, 2H), 4.62 (s, 1H), 4.30, 4.29 (d, CH2 ,2H, JHH = 6.0Hz), 4.05(br. s, NH, 1H). ^{13}C NMR (d-Chloroform, 150MHz) = 146.7, 138.8, 128.9, 128.7, 127.5, 127.4, 127.1, 126.6, 122.1, 113.9 ,54.5, 48.4. MS (ESI/CI): m/z = 217.14, Calculated for $C_{13}H_{12}NCl^+$ $[M]^+$: 217.70 g/mol.

***N*-(4-chlorobenzyl), *N*-(4-chlorophenyl)amine (P5)**

^{1}H NMR (d-chloroform, 600MHz) = 7.39 (d, JHH = 9Hz, 2H, Ar), 7.31 (d, JHH = 9Hz, 2H, Ar), 7.03 (d, JHH = 9Hz, 2H, Ar), 6.57 (d, JHH = 9Hz, 2H, Ar), 4.32 (s, 2H). ^{13}C NMR (d-Chloroform, 150MHz) = 147.2, 138.9, 132.3, 129.0, 128.8, 128.1, 121.8, 114.9, 47.1. MS (ESI/CI): m/z = 253.21, Calculated for $C_{13}H_{11}NCl_2^+$ $[M]^+$: 252.14 g/mol.

***N*-(4-methoxybenzyl), *N*-(4-chlorophenyl)amine (P6)**

^{1}H NMR (d-chloroform, 600MHz) =7.13 (d, JHH = 9Hz, 2H, Ar), 7.04 (d, JHH = 9Hz, 2H, Ar), 6.89 (d, JHH = 9Hz, 2H, Ar), 6.58(d, JHH = 9Hz, 2H, Ar), 4.32 (s, 2H), 3.79 (s, -OCH_3, 3H). ^{13}C NMR (d-Chloroform, 150MHz) = 160.1, 147.3 132.4, 129.0, 128.4, 121.8, 114.9, 114.2, 56.1, 47.1. MS (ESI/CI): m/z = 248.21, Calculated for $C_{14}H_{14}NOCl^+$ $[M]^+$: 247.72 g/mol.

***N*-benzyl, *N*-(4-methoxyphenyl)amine (P7):** ^{1}H NMR (d-chloroform, 600MHz) = 7.37-7.32 (m, Ar, 4H), 7.27-7.25 (t, Ar, 1H), 6.77-6.76 (m, Ar, 2H), 6.59-6.60 (m, Ar, 2H), 4.27 (s, CH2, 2H), 3.73 (s, CH3, 3H).^{13}C NMR ($CDCl_3$, 150MHz) δ (ppm) = 152.2, 142.5, 139.7, 128.6, 127.6, 127.2, 114.9, 114.1, 55.8, 49.3. MS (ESI/CI): m/z = 213.05, Calculated for $C_{14}H_{15}NO^+$ $[M]^+$: 213.28g/mol.

***N*-(4-chlorobenzyl), *N*-(4-methoxyphenyl)amine (P8)**

^{1}H NMR (d-chloroform, 600MHz) =7.38 (d, JHH = 9Hz, 2H, Ar), 7.30(d, JHH = 9Hz, 2H, Ar), 6.76 (d, JHH = 9Hz, 2H, Ar), 6.68(d, JHH = 9Hz, 2H, Ar), 4.31 (s, 2H), 3.78 (s, -OCH_3, 3H). ^{13}C NMR (d-Chloroform, 150MHz) = 152.7, 141.6, 138.9, 132.4, 128.8, 128.1, 115.4, 115.1, 56.1, 47.1. MS (ESI/CI): m/z = 248.61, Calculated for $C_{14}H_{14}NOCl^+$ $[M]^+$:

247.72 g/mol.

***N*-(4-methoxybenzyl), *N*-(4-methoxyphenyl)amine (P9)**

^{1}H NMR (d-chloroform, 600MHz) =7.12 (d, JHH = 9Hz, 2H, Ar), 6.88 (d, JHH = 9Hz, 2H, Ar), 6.78 (d, JHH = 9Hz, 2H, Ar), 6.70(d, JHH = 9Hz, 2H, Ar), 4.32 (s, 2H), 3.90 (s, -OCH$_3$, 3H) , 3.78 (s, -OCH$_3$, 3H). ^{13}C NMR (d-Chloroform, 150MHz) = 160.1, 152.7, 141.6 132.4, 128.4, 115.4, 115.1, 114.2, 57.5, 56.1, 47.1. MS (ESI/CI): m/z = 242.21, Calculated for $C_{15}H_{17}NO_2^+$ $[M]^+$: 243.30g/mol.

***N*-hexylaniline (P10):** ^{1}H NMR (d-chloroform, 600MHz) = 7.17-7.14 (m, Ar, 2H), 6.68-6.66 (m, Ar, 1H), 6.59-6.58 (m, Ar, 2H), 3.57 (br. s, NH, 1H), 3.11-3.0718 (t, 2H), 1.62-1.55 (m, 2H), 1.45-1.39 (m, 2H), 0.96-0.93 (t, 3H). ^{13}C NMR (d-Chloroform, 150MHz) = 148.6, 129.2, 117.7, 112.7, 43.7, 31.7, 28.8, 27.4, 23.3, 134.0. MS (ESI/CI): m/z = 177.17, Calculated for $C_{12}H_{19}N^+$ $[M]^+$: 177.29 g/mol.

***N*-hexyl, *N*-(4-chlorophenyl) amine (P11):**

^{1}H NMR (d-chloroform, 600MHz) =7.03 (d, JHH = 9Hz, 2H, Ar), 6.58 (d, JHH = 9Hz, 2H, Ar), 3.08 (m,1H,), 1.86(m, 2H), 1.60 (m, 2H), 1.25 (m, 2H), 1.12 (m, 2H), ^{13}C NMR (d-Chloroform, 150MHz) = 147.9, 129.1 119.7, 114.5, 52.6, 33.4, 25.9, 24.7. MS (ESI/CI):

m/z = 210.21, Calculated for $C_{12}H_{16}NCl^{+}$ $[M]^{+}$: 209.71 g/mol.

***N*-hexyl, *N*-(4-methoxyphenyl) amine (P12):**

^{1}H NMR (d-chloroform, 600MHz) =6.77 (d, JHH = 9Hz, 2H, Ar), 6.69 (d, JHH = 9Hz, 2H, Ar), 3.79 (s,1H,), 3.37(m, 1H), 1.60 (m, 2H), 1.46 (m, 2H), 1.16 (m, 4H), ^{13}C NMR (d-Chloroform, 150MHz) = 151.8, 142.0 115.3, 114.3, 56.1, 52.6, 33.4, 25.9, 24.7. MS (ESI/CI): m/z = 206.21, Calculated for $C_{13}H_{19}NO^{+}$ $[M]^{+}$: 205.29g/mol.

***N*-Benzylpyrrolidine (P13):**

^{1}H NMR (d-chloroform, 600MHz) =7.23 (m, 5H, Ar), 3.66 (s, 2H), 2.51 (m,4H,), 1.68(m, 4H). ^{13}C NMR (d-Chloroform, 150MHz) = 138.6, 128.7, 128.2, 127.2, 63.3, 54.4, 24.9. MS (ESI/CI): m/z = 162.21, Calculated for $C_{11}H_{15}N^{+}$ $[M]^{+}$: 161.24 g/mol.

***N*-(4-Chlorobenzyl)pyrrolidine (P14):**

^{1}H NMR (d-chloroform, 600MHz) =7.39 (d, JHH = 6Hz, 2H, Ar), 7.32 (d, JHH = 6Hz, 2H, Ar), 3.66 (s,2H,), 2.51(m, 4H), 1.68 (m, 4H). ^{13}C NMR (d-Chloroform, 150MHz) = 137.7, 131.7, 129.8, 128.2, 68.6, 54.4, 24.9. MS (ESI/CI): m/z = 195.21, Calculated for $C_{11}H_{14}NCl^{+}$ $[M]^{+}$: 195.69 g/mol.

_N_-(4-Methoxybenzyl)pyrrolidine (P15):

^{1}H NMR (d-chloroform, 600MHz) =7.13 (d, JHH = 9Hz, 2H, Ar), 6.89 (d, JHH = 9Hz, 2H, Ar), 3.79 (s,3H,), 3.66(s, 2H), 2.51 (m, 4H), 1.68 (m, 4H). ^{13}C NMR (d-Chloroform, 150MHz) = 160.4, 130.2, 129.1, 114.1, 63.6, 56.1, 54.4, 24.9. MS (ESI/CI): m/z = 192.21, Calculated for $C_{12}H_{17}NO^+$ $[M]^+$: 191.27 g/mol.

Benzylpiperazine (P16): ^{1}H NMR ($CDCl_3$, 600MHz) δ (ppm) = 7.29-7.22 (m, Ar, 5H), 3.56-3.40 (m, 4H), 2.89-2.86 (m, 4H), 2.41 (br. s, NH , 1H). ^{13}C NMR ($CDCl_3$, 150MHz) δ (ppm) = 137.8, 129.2, 128.1, 127.0, 64.0, 54.1, 45.8. HRMS (ESI/APCI): *m/z* = 177.2642 g/mol, calc'd. for $C_{11}H_{17}N_2^+$ $[M]^+$: 177.2644g/mol.

(4-Chlorobenzyl) piperazine (P17):

^{1}H NMR (d-chloroform, 600MHz) =7.40 (d, JHH = 9Hz, 2H, Ar), 7.32 (d, JHH = 9Hz, 2H, Ar), 3.66 (s,2H,), 2.68 (m, 4H), 2.34 (m, 4H). ^{13}C NMR (d-Chloroform, 150MHz) = 137.7, 131.7, 129.4, 128.3, 63.4, 53.5, 46.7. MS (ESI/CI): m/z = 210.21, Calculated for $C_{11}H_{14}N_2Cl^+$ $[M]^+$: 209.69 g/mol.

(4-Methoxybenzyl) piperazine (P18)

^{1}H NMR (d-chloroform, 600MHz) =7.13 (d, JHH = 9Hz, 2H, Ar), 6.89 (d, JHH = 9Hz, 2H,

Ar), 3.79 (s,3H,), 3.66 (s, 2H), 2.68 (m, 4H), 2.34 (m, 4H). ^{13}C NMR (d-Chloroform, 150MHz) = 160.4, 130.2, 129.1, 114.35, 68.4, 56.1, 52.5, 45.7. MS (ESI/CI): m/z = 204.21, Calculated for $C_{12}H_{17}N_2O^+$ $[M]^+$: 205.27 g/mol.

***N*-phenylpyrrolidine (P19):** ^{1}H NMR (d-chloroform, 600MHz) = 7.26 (m, 2H), 7.12 (m, 1H), 6.84(m, 1H), 3.19–3.18 (m, 4H), 2.33 (s, 3H), 1.94-1.92 (m,4H). ^{13}C NMR (d-Chloroform, 150MHz) = 148.6, 129.7, 115.8, 111.97, 47.9, 26.0. MS (ESI/CI): m/z =147.13, Calculated for C13H13N: 147.23 g/mol.

***N*-phenylpiperidine (P20):** ^{1}H NMR (d-chloroform, 600MHz) = 7.45 (t, 2H, J=7.4 Hz), 6.99 (d, 2H, J=7.8 Hz), 6.73 (t, 1H, J=7.3 Hz), 3.36 (t, 4H, J=6.7 Hz), 1.81 (m, 4H), 1.62 (t, 2H, J=4.8 Hz). ^{13}C NMR ($CDCl_3$, 150MHz) δ (ppm) = 152.6, 129.9, 119.7, 117.3, 60.12, 26.6, 24.9. MS (ESI/CI): m/z = 161.12, Calculated for $C_{11}H_{15}N^+$ $[M]^+$: 161.25 g/mol.

***N*-phenylcycloheptamine (P21)**

^{1}H NMR (d-chloroform, 600MHz) =7.30 (m, 2H, Ar), 6.91 (m, 2H, Ar), 6.71 (m, 1H, Ar), 3.36 (m, 4H), 1.78(m, 4H), 1.66 (m, 4H). ^{13}C NMR (d-Chloroform, 150MHz) = 150.1, 130.3, 117.3, 114.4, 51.3, 27.6, 27.5. MS (ESI/CI): m/z = 174.81, Calculated for

$C_{12}H_{17}N^+$ $[M]^+$: 175.27 g/mol.

N-Methyl-N'-phenylpiperazine (P22): ^{1}H NMR (d-chloroform, 600MHz) = 8.21 (dd, 1H),7.66 (ddd, 1H), 6.88 – 6.78 (m, 2H), 3.74 –3.66 (m, 4H), 3.22 – 3.13 (m, 4H). ^{13}C NMR (d-Chloroform, 150MHz) = 160.4, 147.9, 137.6, 110.1, 107.2, 46.1, 45.8. MS (ESI/CI): m/z = 177.23, Calculated for $C_{11}H_{16}N_2^+$ $[M]^+$: 176.25 g/mol.

N-(4-Chlorophenyl), N'-methylpiperazine (**P23**): ^{1}H NMR (d-chloroform, 600MHz) =7.13 (d, JHH = 9Hz, 2H, Ar), 6.72 (d, JHH = 9Hz, 2H, Ar), 3.44 (m,4H,), 2.35 (m, 4H), 2.20 (s, 3H. ^{13}C NMR (d-Chloroform, 150MHz) = 149.0, 129.5, 123.1, 116.8, 52.7, 50.3, 46.1. MS (ESI/CI): m/z = 210.92, Calculated for $C_{11}H_{15}N_2Cl^+$ $[M]^+$: 210.70g/mol.

N-(4-Methoxyphenyl), N'-methylpiperazine (**P24**):^{1}H NMR (d-chloroform, 600MHz) =6.74 (d, JHH = 9Hz, 2H, Ar), 6.65 (d, JHH = 9Hz, 2H, Ar), 3.79 (s,3H,), 3.41(m, 4H), 2.35 (m, 4H), 2.20 (s, 3H). ^{13}C NMR (d-Chloroform, 150MHz) = 152.6, 143.4, 117.5, 115.5, 56.1, 52.6, 50.1, 46.1. MS (ESI/CI): m/z = 205.92, Calculated for $C_{12}H_{18}N_2O^+$ $[M]^+$: 206.28 g/mol.

VI. The Atomic Coordinates of **Cat. 1~7**

Table S6. The Atomic Coordinates (angstroms) of **Cat. 1**

Element	Coordinates (Angstroms)		
	X	Y	Z
Ir	0.765	0.7634	0.6258
P	0.6883	0.7259	0.8096
Cl(1)	0.9909	0.7284	0.6273
N(1)	0.7717	0.9187	0.6236
N(2)	0.7515	0.6147	0.6045
C(1)	0.7221	0.9773	0.7042
C(2)	0.7366	1.0795	0.6972
C(3)	0.8057	1.1259	0.6032
C(4)	0.8507	1.0683	0.5194
C(5)	0.8307	0.9655	0.5291
C(6)	0.8667	0.8996	0.4436
C(7)	0.9211	0.9357	0.3408
C(8)	0.9509	0.8693	0.2639
C(9)	0.9259	0.7678	0.2891
C(10)	0.8709	0.7322	0.3908
C(11)	0.8403	0.7974	0.4706
C(12)	0.8456	0.5381	0.596
C(13)	0.8314	0.4409	0.5781
C(14)	0.7156	0.4199	0.5703
C(15)	0.6181	0.498	0.5798
C(16)	0.6371	0.5958	0.596
C(17)	0.5445	0.686	0.6023
C(18)	0.4203	0.6827	0.5994
C(19)	0.34	0.7719	0.6022
C(20)	0.3845	0.8647	0.6061
C(21)	0.5077	0.868	0.6102
C(22)	0.5891	0.7786	0.6118
C(23)	0.7911	0.752	0.8821
C(24)	0.8763	0.8272	0.8458
C(25)	0.9488	0.85	0.9033
C(26)	0.939	0.7974	0.9979

C(27)	0.8552	0.723	1.0351
C(28)	0.7818	0.6998	0.9783
C(29)	0.6707	0.5895	0.8416
C(30)	0.7795	0.5227	0.8379
C(31)	0.7732	0.4185	0.8545
C(32)	0.6577	0.3779	0.8731
C(33)	0.5508	0.4426	0.8743
C(34)	0.5559	0.5477	0.8601
C(35)	0.5323	0.7898	0.8829
C(36)	0.5192	0.8484	0.9643
C(37)	0.402	0.9006	1.0151
C(38)	0.2955	0.8935	0.9865
C(39)	0.3063	0.8351	0.9066
C(40)	0.4236	0.7845	0.8538
Cl(2)	0.3794	1.0782	0.786
Cl(3)	0.4363	1.2851	0.706
C(41)	0.4157	1.1654	0.6765
Cl(4)	0.8422	0.5053	1.2666
Cl(5)	0.8084	0.4252	1.0898
C(42)	0.8725	0.4002	1.1902

Table S7. The Atomic Coordinates (angstroms) of **Cat. 2**

Element	Coordinates (Angstroms)		
	X	Y	Z
Ir	0.8964	0.8514	0.8235
Cl	0.9558	1.007	0.757
P	1.0623	0.7218	0.7016
O(1)	1.2618	0.2354	0.824
O(2)	1.5173	0.7842	0.5464
O(3)	0.864	0.7686	0.3754
N(1)	0.9888	0.8191	0.9234
N(2)	0.7804	0.8932	0.7414
C(1)	1.0997	0.7353	0.9208
C(2)	1.1567	0.7247	0.987
C(3)	1.1004	0.8008	1.0607
C(4)	0.9867	0.8836	1.067
C(5)	0.9294	0.892	0.9989
C(6)	0.8055	0.9717	1.002
C(7)	0.7294	1.0521	1.0752
C(8)	0.6112	1.118	1.0781
C(9)	0.5673	1.1062	1.0079
C(10)	0.6422	1.0305	0.934
C(11)	0.7641	0.9621	0.928
C(12)	0.7548	0.9813	0.6837
C(13)	0.6752	1.0053	0.63
C(14)	0.6223	0.9342	0.6364
C(15)	0.6464	0.844	0.6974
C(16)	0.7258	0.8233	0.7513
C(17)	0.7538	0.7347	0.8214
C(18)	0.708	0.6534	0.8419
C(19)	0.7345	0.5764	0.9117
C(20)	0.8042	0.5804	0.9646
C(21)	0.8515	0.659	0.9459
C(22)	0.8297	0.7366	0.8721
C(23)	1.1298	0.5714	0.7327
C(24)	1.0568	0.5122	0.7655
C(25)	1.1051	0.4003	0.7935

C(26)	1.2241	0.3457	0.7941
C(27)	1.2966	0.402	0.7639
C(28)	1.2492	0.5127	0.7334
C(29)	1.1986	0.7465	0.6504
C(30)	1.2769	0.7006	0.5626
C(31)	1.383	0.7117	0.5244
C(32)	1.4144	0.7682	0.5748
C(33)	1.3392	0.8136	0.6628
C(34)	1.2315	0.8044	0.6996
C(35)	1.0152	0.7264	0.5965
C(36)	0.9982	0.8207	0.5385
C(37)	0.9506	0.8387	0.4626
C(38)	0.9152	0.7594	0.4455
C(39)	0.933	0.6648	0.502
C(40)	0.982	0.6482	0.5755
C(41)	1.378	0.1789	0.8403
C(42)	1.6002	0.7363	0.456
C(43)	0.8274	0.8713	0.3266
C(44)	0.4886	0.4874	0.8491
C(45)	0.5581	0.3703	0.8623
C(46)	0.6621	0.2973	0.804
C(47)	0.7201	0.3369	0.7246
C(48)	0.6563	0.4548	0.7055
C(49)	0.5508	0.5147	0.7673
C(50)	0.7246	0.4914	0.6265

Table S8. The Atomic Coordinates (angstroms) of **Cat. 3**

Element	Coordinates (Angstroms)		
	X	Y	Z
Ir	0.725	0.7304	0.8709
Cl(1)	0.5027	0.7644	0.8677
P	0.7921	0.7724	0.6941
F(1)	0.4696	0.6998	0.4418
F(2)	0.8192	1.2165	0.6362
F(3)	1.2715	0.5571	0.481
N(1)	0.7196	0.5765	0.8666
N(2)	0.7384	0.8774	0.8988
C(1)	0.761	0.5227	0.7858
C(2)	0.751	0.4193	0.7881
C(3)	0.6968	0.3698	0.8771
C(4)	0.6592	0.4222	0.9597
C(5)	0.6728	0.5266	0.9558
C(6)	0.6423	0.5881	1.0406
C(7)	0.596	0.5496	1.1374
C(8)	0.5678	0.6125	1.2152
C(9)	0.5873	0.7146	1.1946
C(10)	0.633	0.7524	1.0984
C(11)	0.662	0.691	1.0188
C(12)	0.897	0.7162	0.8884
C(13)	0.978	0.6261	0.8913
C(14)	1.096	0.6303	0.8992
C(15)	1.1415	0.7207	0.9039
C(16)	1.0651	0.8092	0.9048
C(17)	0.9417	0.808	0.8997
C(18)	0.8504	0.8975	0.9071
C(19)	0.871	0.9934	0.9236
C(20)	0.7755	1.0705	0.9352
C(21)	0.6604	1.0504	0.929
C(22)	0.6446	0.9553	0.9078
C(23)	0.6891	0.7517	0.6196
C(24)	0.6053	0.6793	0.6471
C(25)	0.5325	0.6612	0.5866

C(26)	0.5437	0.7174	0.4991
C(27)	0.6239	0.7881	0.4685
C(28)	0.6973	0.8076	0.526
C(29)	0.8088	0.9079	0.6668
C(30)	0.7014	0.9729	0.6796
C(31)	0.7047	1.0766	0.6691
C(32)	0.8141	1.1132	0.645
C(33)	0.9239	1.0543	0.6286
C(34)	0.9224	0.9486	0.6397
C(35)	0.9413	0.7099	0.6244
C(36)	0.9509	0.6576	0.5413
C(37)	1.0621	0.6076	0.4932
C(38)	1.1605	0.6081	0.527
C(39)	1.1591	0.6576	0.6083
C(40)	1.0454	0.7105	0.658
C(41)	1.6527	1.0955	0.3002
Cl(2)	1.7119	1.0798	0.3969
Cl(3)	1.6722	0.9931	0.2304
C(42)	0.9263	0.6755	0.1729
Cl(4)	0.9438	0.7938	0.1981
Cl(5)	0.8887	0.594	0.2764

Table S9. The Atomic Coordinates (angstroms) of **Cat. 4**

Element	Coordinates (Angstroms)		
	X	Y	Z
Ir	0.511	0.7363	0.7205
Cl(1)	0.561	0.5513	0.7569
P(1)	0.315	0.7038	0.822
O(1)	0.5651	0.9519	0.8824
O(2)	0.5915	0.6509	0.4673
O(3)	-0.0817	0.9722	0.7878
O(4)	0.1251	0.2567	0.8572
O(5)	0.3272	0.7889	1.175
N(1)	0.5561	0.8024	0.8176
N(2)	0.4954	0.6897	0.6055
C(1)	0.482	0.8949	0.6936
C(2)	0.4478	0.9425	0.6282
C(3)	0.4283	1.0527	0.6249
C(4)	0.4419	1.1172	0.6866
C(5)	0.4785	1.0735	0.7509
C(6)	0.5005	0.9622	0.7544
C(7)	0.5393	0.9057	0.8178
C(8)	0.5984	0.7737	0.884
C(9)	0.6335	0.6772	0.9122
C(10)	0.6704	0.6785	0.9833
C(11)	0.6689	0.771	1.0258
C(12)	0.6348	0.8684	0.9973
C(13)	0.6022	0.8652	0.925
C(14)	0.6829	0.7521	0.6363
C(15)	0.779	0.7845	0.6565
C(16)	0.8868	0.7846	0.592
C(17)	0.9056	0.7458	0.5118
C(18)	0.8118	0.7105	0.4927
C(19)	0.7032	0.7136	0.5553
C(20)	0.5976	0.6821	0.5427
C(21)	0.4137	0.6636	0.5679
C(22)	0.295	0.6601	0.5995
C(23)	0.2409	0.6253	0.5441

C(24)	0.3049	0.5957	0.46
C(25)	0.4227	0.6005	0.4284
C(26)	0.4753	0.6361	0.4833
C(27)	0.1929	0.7858	0.8114
C(28)	0.2064	0.8969	0.7964
C(29)	0.1172	0.9616	0.7881
C(30)	0.0118	0.9161	0.7953
C(31)	-0.0631	1.081	0.7537
C(32)	-0.0037	0.8068	0.8113
C(33)	0.0854	0.741	0.8194
C(34)	0.2578	0.5665	0.8354
C(35)	0.1726	0.5284	0.9131
C(36)	0.126	0.4253	0.9227
C(37)	0.1634	0.3594	0.8535
C(38)	0.0363	0.2166	0.9351
C(39)	0.2472	0.3963	0.776
C(40)	0.2945	0.498	0.7678
C(41)	0.3106	0.7262	0.9331
C(42)	0.2761	0.8246	0.9711
C(43)	0.2823	0.8422	1.0513
C(44)	0.3235	0.7635	1.0962
C(45)	0.3597	0.666	1.0594
C(46)	0.3534	0.6497	0.9785
C(47)	0.3606	0.7064	1.2258
C(48)	0.2499	0.952	0.4606
Cl(2)	0.1969	0.89	0.401
Cl(3)	0.1779	0.9466	0.5697
C(49)	1.1155	0.4654	0.277
Cl(4)	1.0567	0.5453	0.3624
Cl(5)	1.0936	0.5062	0.184

Table S10. The Atomic Coordinates (angstroms) of **Cat. 5**

Element	Coordinates (Angstroms)		
	X	Y	Z
Ir	-0.0006	0.199	0.2292
Cl(1)	0.076	0.2524	0.3393
Cl(2)	0.4901	0.0055	0.1231
Cl(3)	0.2126	0.4665	0.0853
P	-0.1923	0.3377	0.2416
O(1)	0.0945	-0.0768	0.3828
O(2)	-0.0768	0.1732	-0.0091
O(3)	-0.2687	0.7823	0.2496
O(4)	-0.49	0.308	-0.0233
O(5)	-0.4317	0.3383	0.5706
N(1)	-0.0148	0.076	0.3176
N(2)	-0.0537	0.1517	0.1261
C(1)	0.1704	0.0802	0.2217
C(2)	0.2637	0.0813	0.1735
C(3)	0.3734	0.0027	0.185
C(4)	0.3958	-0.0778	0.2441
C(5)	0.3041	-0.0805	0.2932
C(6)	0.1917	-0.0016	0.2815
C(7)	0.0901	0.001	0.3281
C(8)	-0.0885	0.0457	0.3704
C(9)	-0.0193	-0.0482	0.4105
C(10)	-0.0609	-0.1024	0.4682
C(11)	-0.1796	-0.0575	0.4853
C(12)	-0.2516	0.0365	0.4446
C(13)	-0.2084	0.0888	0.3867
C(14)	0.0418	0.2848	0.1388
C(15)	0.1038	0.3469	0.1448
C(16)	0.1359	0.3901	0.0734
C(17)	0.111	0.3753	-0.0047
C(18)	0.0513	0.3148	-0.0134
C(19)	0.0187	0.2695	0.0576
C(20)	-0.0364	0.1985	0.0576
C(21)	-0.1071	0.0887	0.1065

C(22)	-0.1227	0.1041	0.0214
C(23)	-0.1772	0.0565	-0.0209
C(24)	-0.2153	-0.0094	0.0269
C(25)	-0.1999	-0.026	0.1109
C(26)	-0.1435	0.0218	0.1527
C(27)	-0.2064	0.4732	0.2322
C(28)	-0.1215	0.4974	0.2613
C(29)	-0.1382	0.5994	0.2681
C(30)	-0.242	0.6786	0.2441
C(31)	-0.3245	0.6561	0.2127
C(32)	-0.312	0.5554	0.2073
C(33)	-0.2036	0.8107	0.3026
C(34)	-0.2884	0.3341	0.1639
C(35)	-0.2982	0.3863	0.0863
C(36)	-0.3652	0.3765	0.0256
C(37)	-0.4252	0.3128	0.0396
C(38)	-0.4145	0.2579	0.1142
C(39)	-0.3464	0.2692	0.1743
C(40)	-0.5448	0.238	-0.0113
C(41)	-0.2734	0.3434	0.3405
C(42)	-0.3935	0.3765	0.3464
C(43)	-0.4494	0.3758	0.4229
C(44)	-0.3854	0.3445	0.4932
C(45)	-0.2659	0.3153	0.4885
C(46)	-0.2118	0.3145	0.4135
C(47)	-0.5547	0.3649	0.5782

Table S11. The Atomic Coordinates (angstroms) of **Cat. 6**

Element	Coordinates (Angstroms)		
	X	Y	Z
Ir	-0.4923	0.2622	0.5172
Cl	-0.6112	0.3043	0.5079
P	-0.4891	0.2389	0.4174
F(1)	-0.5436	0.3746	0.7576
F(2)	-0.5403	0.1875	0.644
F(3)	-0.2251	0.1061	0.5802
F(4)	-0.3221	0.3098	0.5361
O(1)	-0.446	0.4514	0.5655
O(2)	-0.4938	0.0655	0.5547
N(1)	-0.4557	0.3583	0.516
N(2)	-0.5237	0.1676	0.5307
C(1)	-0.4237	0.4052	0.4812
C(2)	-0.3968	0.4037	0.4276
C(3)	-0.3696	0.4607	0.4058
C(4)	-0.3683	0.5191	0.4385
C(5)	-0.3926	0.5206	0.4931
C(6)	-0.4194	0.4627	0.5121
C(7)	-0.4664	0.3882	0.5644
C(8)	-0.4929	0.3549	0.613
C(9)	-0.5051	0.3847	0.6645
C(10)	-0.5293	0.347	0.7067
C(11)	-0.5424	0.281	0.6999
C(12)	-0.5287	0.2524	0.6478
C(13)	-0.5052	0.2867	0.6017
C(14)	-0.5855	0.13	0.5338
C(15)	-0.6558	0.1442	0.5241
C(16)	-0.7017	0.0924	0.5298
C(17)	-0.6804	0.0297	0.5452
C(18)	-0.6115	0.0151	0.5549
C(19)	-0.5657	0.067	0.5488
C(20)	-0.4732	0.1272	0.5434
C(21)	-0.4028	0.1507	0.5474
C(22)	-0.3462	0.1113	0.5616

C(23)	-0.2828	0.1422	0.566
C(24)	-0.2744	0.2088	0.5584
C(25)	-0.3334	0.2447	0.5445
C(26)	-0.3989	0.2192	0.537
C(27)	-0.5047	0.3066	0.3683
C(28)	-0.5492	0.3565	0.3817
C(29)	-0.5598	0.4091	0.345
C(30)	-0.5258	0.4114	0.2945
C(31)	-0.481	0.3614	0.2803
C(32)	-0.4712	0.3093	0.3163
C(34)	-0.4089	0.2008	0.3898
C(35)	-0.4094	0.1546	0.3474
C(36)	-0.3465	0.1281	0.328
C(37)	-0.2839	0.1478	0.3501
C(38)	-0.2832	0.195	0.3901
C(39)	-0.3449	0.2216	0.4101
C(41)	-0.5573	0.1803	0.3984
C(42)	-0.6216	0.2006	0.3782
C(43)	-0.6744	0.155	0.3683
C(44)	-0.6626	0.0899	0.3767
C(45)	-0.5994	0.0689	0.3954
C(46)	-0.5479	0.113	0.4071
O(6)	-0.2601	0.2966	0.2326
C(48)	-0.3277	0.2685	0.2081
O(7)	-0.2796	0.3926	0.2342
O(8)	-0.2157	0.363	0.2925
O(3)	-0.5484	0.466	0.2586
O(4)	-0.2114	0.1296	0.3373
O(5)	-0.7096	0.0383	0.3721
C(33)	-0.5156	0.4656	0.206
C(40)	-0.2127	0.0799	0.2918
C(47)	-0.7839	0.0557	0.3716
O(3')	-0.526	0.465	0.2666
O(4')	-0.2312	0.1171	0.328
O(5')	-0.723	0.0504	0.3594
C(33')	-0.481	0.47	0.208
C(40')	-0.1648	0.1397	0.3239
C(47')	-0.79	0.075	0.35

Table S12. The Atomic Coordinates (angstroms) of **Cat. 7**

Element	Coordinates (Angstroms)		
	X	Y	Z
Ir	0.6904	0.9199	0.7672
Cl(1)	0.5279	0.932	0.7012
Cl(2)	0.5174	0.8252	0.3538
Cl(3)	0.8799	1.3686	1.1086
P	0.816	1.1163	0.7572
O(1)	0.8313	0.7762	0.5767
O(2)	0.5436	0.8778	0.9966
O(3)	0.858	1.0872	0.3739
O(4)	1.2978	1.36	0.9435
O(5)	0.6406	1.4579	0.8615
N(1)	0.7265	0.8541	0.6473
N(2)	0.6503	0.9534	0.8942
C(1)	0.6947	0.828	0.5568
C(2)	0.6181	0.8416	0.509
C(3)	0.6118	0.8096	0.4182
C(4)	0.6815	0.7671	0.3761
C(5)	0.7558	0.7505	0.4225
C(6)	0.7606	0.7811	0.5125
C(7)	0.8045	0.8202	0.6554
C(8)	0.8559	0.8331	0.7397
C(9)	0.9374	0.7994	0.7538
C(10)	0.9762	0.8156	0.84
C(11)	0.9347	0.8636	0.9103
C(12)	0.858	0.9011	0.8946
C(13)	0.8138	0.8864	0.809
C(14)	0.6864	1.0425	0.9713
C(15)	0.771	1.1583	0.9954
C(16)	0.7788	1.2226	1.0781
C(17)	0.7054	1.1749	1.1382
C(18)	0.6258	1.0607	1.1186
C(19)	0.6182	0.9957	1.0339
C(20)	0.5681	0.8593	0.9116
C(21)	0.5182	0.7489	0.85

C(22)	0.4294	0.646	0.8626
C(23)	0.3931	0.5444	0.7948
C(24)	0.4399	0.5469	0.7191
C(25)	0.5287	0.6516	0.7063
C(26)	0.57	0.7541	0.7713
C(27)	0.8352	1.1175	0.642
C(28)	0.9206	1.1014	0.5991
C(29)	0.9244	1.0906	0.5099
C(30)	0.8453	1.0993	0.4604
C(31)	0.7599	1.1173	0.5008
C(32)	0.7545	1.1251	0.59
C(33)	0.7778	1.0909	0.3177
C(34)	0.9627	1.1919	0.8148
C(35)	1.0127	1.3036	0.8723
C(36)	1.1212	1.3535	0.9139
C(37)	1.1874	1.2966	0.8988
C(38)	1.1395	1.1846	0.8422
C(39)	1.0295	1.1334	0.802
C(40)	1.3677	1.3101	0.9357
C(41)	0.7667	1.2244	0.7916
C(42)	0.8211	1.3307	0.7713
C(43)	0.7848	1.4131	0.7958
C(44)	0.6878	1.3871	0.8372
C(45)	0.6334	1.2813	0.8589
C(46)	0.6708	1.2001	0.8351
C(47)	0.6939	1.5701	0.8419
C(48)	0.662	1.396	0.454
Cl(4)	0.7296	1.3613	0.5318
Cl(5)	0.6172	1.4754	0.538
O(6')	0.888	1.568	0.324
O(6)	0.924	1.564	0.397
